# Mitoxantrone‐Encapsulated ZIF‐8 Enhances Chemo‐Immunotherapy via Amplified Immunogenic Cell Death

**DOI:** 10.1002/advs.202501542

**Published:** 2025-02-14

**Authors:** Junhong Li, Wenxing Lv, Ziwei Han, Yike Li, Jinqi Deng, Yanjuan Huang, Shuo Wan, Jiashu Sun, Bo Dai

**Affiliations:** ^1^ Department of Urology Fudan University Shanghai Cancer Center Shanghai 200032 P. R. China; ^2^ Department of Oncology Shanghai Medical College Fudan University Shanghai 200032 P. R. China; ^3^ Beijing Engineering Research Center for BioNanotechnology CAS Key Laboratory of Standardization and Measurement for Nanotechnology National Center for Nanoscience and Technology Beijing 100190 P. R. China; ^4^ School of Future Technology University of Chinese Academy of Sciences Beijing 100049 P. R. China; ^5^ Foundation for Applied Molecular Evolution Alachua Florida 32615 US

**Keywords:** chemo‐immunotherapy, immunogenic cell death, prostate cancer, pyroptosis, zeolitic imidazolate framework‐8

## Abstract

Chemo‐immunotherapy, combining systemic chemotherapeutic drugs and immune checkpoint blockers, is a promising paradigm in cancer treatment. However, challenges such as limited induction of immune responses and systemic immune toxicity have hindered its clinical applications. Here, a zeolite imidazolate framework‐8 (ZIF‐8) that encapsulates mitoxantrone (MIT), an immune cell death (ICD)‐inducing chemotherapeutic agent (MIT@ZIF‐8), is synthesized using a one‐pot aqueous‐phase process. ZIF‐8 serves as a dual‐functional nanomaterial for chemo‐immunotherapy: a carrier to enhance tumor uptake of MIT for improved chemotherapy efficacy, and a pyroptosis inducer to amplify MIT‐induced ICD for augmented anti‐tumor immune responses. As a result, in vivo administration of MIT@ZIF‐8 markedly inhibits tumor growth in both immunologically “hot” colon cancer and immunologically “cold” prostate cancer. Moreover, MIT@ZIF‐8 treatment increases the abundance of cytotoxic CD8^+^ T cells and reduces the amount of immunosuppressive regulatory T cells in tumors, thereby enhancing anti‐tumor immunity and sensitizing prostate cancer to anti‐CTLA‐4 immunotherapy. In summary, MIT@ZIF‐8 offers a highly translational approach for chemo‐immunotherapy.

## Graphical abstract

Mitoxantrone (MIT)‐encapsulated zeolite imidazolate framework‐8 nanoparticles (MIT@ZIF‐8) have been designed and synthesized for chemo‐immunotherapy. This regimen enhances MIT‐induced immunogenic cell death via zinc ions‐mediated pyroptosis within tumor, and reduces immunotoxicity in tumor‐draining lymph nodes. The activated anti‐tumor immune microenvironment by MIT@ZIF‐8 improves chemo‐immunotherapy outcomes in various cancers. Furthermore, MIT@ZIF‐8 sensitizes immunologically “cold” prostate cancer to anti‐CTLA‐4 immunotherapy.

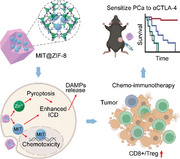

## 1 Introduction

Chemo‐immunotherapy is a promising therapeutic approach for cancer treatment, which enhances the anti‐tumor efficacy via the combination of cytotoxic effects caused by chemotherapy and immune responses induced by immunotherapy.^[^ ^1^ ^]^ Chemotherapy has traditionally been viewed as incompatible with immunotherapy due to its immunosuppressive characteristics. However, accumulating evidence shows that certain chemotherapeutic drugs, such as mitoxantrone (MIT), doxorubicin, and oxaliplatin, can elicit immunogenic cell death (ICD) to stimulate the anti‐tumor immune response.^[^ ^2^ ^]^ ICD is a form of regulated cell death that triggers both innate and adaptive immune systems through the release of damage‐associated molecular patterns (DAMPs), including high mobility group box 1 (HMGB1) and calreticulin (CRT).^[^ ^3^ ^]^ These DAMPs facilitate the maturation of antigen‐presenting cells to activate CD8^+^ T cells for tumor eradication.^[^ ^4^ ^]^ The co‐administration of chemotherapeutic regimens with immune checkpoint blockers (ICB) has exhibited remarkable benefits for treating immunogenic tumors, including urothelial carcinoma,^[^ ^5^ ^]^ non‐small cell lung cancer,^[^ ^6^ ^]^ gastric cancer,^[^ ^7^ ^]^ and triple‐negative breast cancer, in recent clinical trials.^[^ ^8^ ^]^ However, for immunologically “cold” cancers such as prostate cancer, the efficacy of chemo‐immunotherapy is often limited by the weak immune responses against the tumor.^[^ ^9^ ^]^ Additionally, the off‐tumor toxicity associated with systemic chemotherapy may broadly deplete healthy immune cells, potentially compromising the overall immune function.^[^ ^10^ ^]^

Nanomedicine has advanced a variety of modalities for cancer treatment, including chemotherapy, immunotherapy, and gene therapy.^[^ ^11^ ^]^ Among the myriad of nanomaterials, zeolitic imidazolate framework‐8 (ZIF‐8), a metal–organic framework composed of Zn^2+^ and 2‐methylimidazole (2‐MIm), is particularly advantageous due to its large surface area, efficient loading of therapeutic agents, pH‐responsive biodegradation, and good biocompatibility.^[^ ^12^ ^]^ These properties enable ZIF‐8 to effectively deliver drugs, photothermal conversion agents, and photosensitizers to tumor sites, thus enhancing therapeutic outcomes across a spectrum of cancer treatments.^[^ ^12^, ^13^ ^]^ In addition to its role as a carrier, the release of Zn^2+^ ions from ZIF‐8 can induce caspase‐1/Gasdermin‐D (GSDMD)‐dependent pyroptosis,^[^ ^14^ ^]^ a form of ICD that promotes anti‐tumor immune responses.^[^ ^4^, ^15^ ^]^ Encapsulating a mitochondrial depolarizing agent within ZIF‐8 further intensifies the pyroptosis process, leading to the suppression of tumor growth in 4T1 tumor‐bearing mice.^[^ ^14a^ ^]^ Despite these promising developments, further investigation is needed to fully realize the therapeutic potential of ZIF‐8 in chemo‐immunotherapy.

**Scheme 1:**
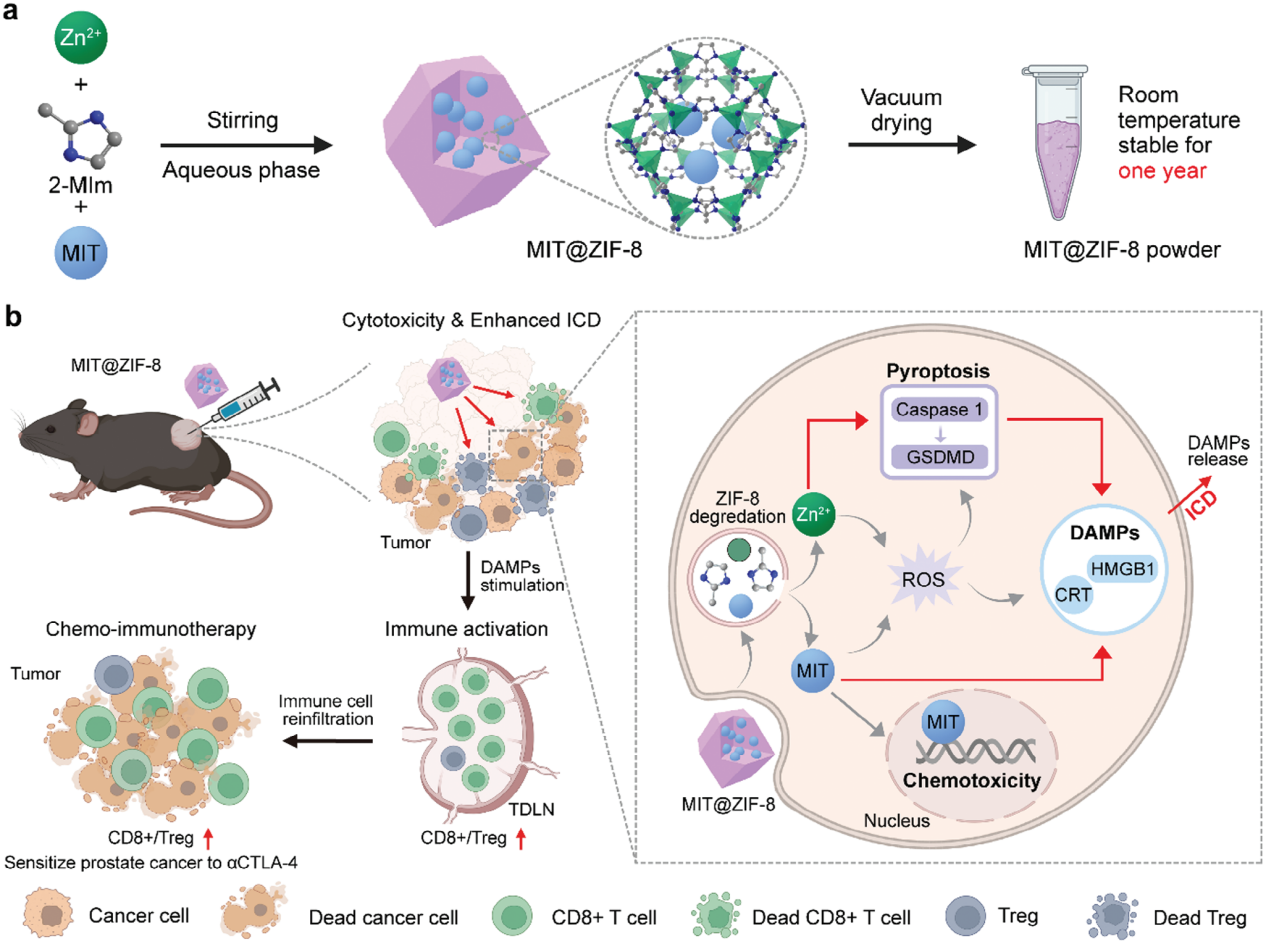
a) Schematic of the design and synthesis of MIT@ZIF‐8 nanoparticles for enhanced chemo‐immunotherapy. b) MIT@ZIF‐8 effectively amplifies ICD through increased tumor uptake of MIT and pyroptosis triggered by Zn^2+^ ions, augmenting chemo‐immunotherapy for both immunologically “hot” and “cold” cancers and sensitizing prostate cancer to anti‐CTLA‐4 immunotherapy.

Here, we propose that MIT‐encapsulated ZIF‐8 nanoparticles (MIT@ZIF‐8) synthesized via a one‐pot aqueous‐phase process can augment the efficacy of chemo‐immunotherapy (**Scheme** **1**). MIT@ZIF‐8 increases the tumor uptake of MIT to enhance its localized cytotoxic effect, while reducing the immune toxicity of MIT to tumor‐draining lymph nodes (TLDNs). Additionally, the Zn^2+^ ions released from MIT@ZIF‐8 trigger pyroptosis, thus augmenting ICD induced by MIT. This process facilitates the release of DAMPs for eliciting a potent anti‐tumor immune response. We show that in vivo administration of MIT@ZIF‐8 effectively inhibits tumor growth in both immunologically “hot” colon cancer and immunologically “cold” prostate cancer. Remarkably, MIT@ZIF‐8 treatment reconfigures the anti‐tumor microenvironment of prostate cancer, rendering it responsive to anti‐CTLA‐4 immunotherapy.

## 2 Results and Discussion

### 2.1 One‐Pot Aqueous‐Phase Synthesis of MIT@ZIF‐8 Nanoparticles

**Figure 1:**
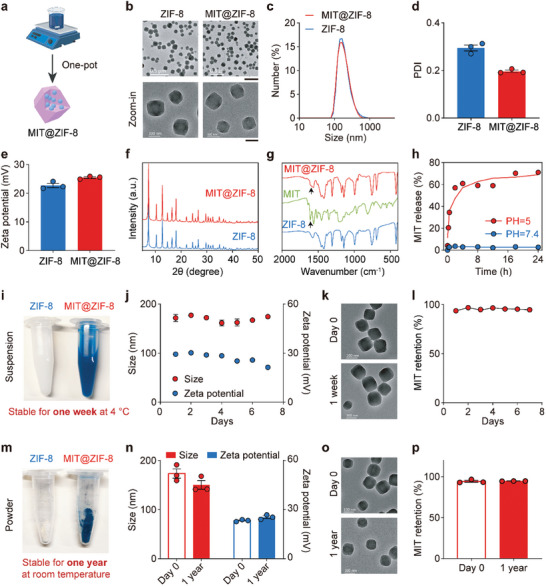
Characterization of MIT@ZIF‐8 nanoparticles. a) Schematic illustration of one‐pot aqueous‐phase synthesis of MIT@ZIF‐8 nanoparticles. b) Representative TEM images displaying ZIF‐8 and MIT@ZIF‐8 nanoparticles. Scale bars are 0.5 µm (top) and 100 nm (bottom), respectively. c) DLS analysis of the size distribution and d) PDI of ZIF‐8 and MIT@ZIF‐8. e) Zeta potentials of ZIF‐8 and MIT@ZIF‐8 determined by DLS. f) XRD patterns of MIT@ZIF‐8 and ZIF‐8. g) FTIR spectra of ZIF‐8, MIT, and MIT@ZIF‐8, with arrows indicating the C=O bond stretching vibrations of MIT. h) MIT release profiles of MIT@ZIF‐8 nanoparticles in PBS at pH 7.4 and pH 5.0. Some error bars are too small to be visible. i) Photographs of ZIF‐8 and MIT@ZIF‐8 nanoparticles stored in aqueous solution at 4 °C. j) DLS measurement of size and zeta potential of MIT@ZIF‐8 nanoparticles stored in aqueous solution at 4 °C for 7 days. k) TEM characterization of MIT@ZIF‐8 stored in aqueous solution at 4 °C on day 0 and on day 7. l) MIT retention level in MIT@ZIF‐8 nanoparticles in aqueous solution at 4 °C for 7 days. Some error bars are too small to be visible. m) Photographs of ZIF‐8 and MIT@ZIF‐8 nanoparticles as dry powders after one‐year storage at room temperature. n) DLS measurement of size distribution and zeta potential of MIT@ZIF‐8 immediately after synthesis (day 0) and the resuspended MIT@ZIF‐8 after one‐year storage (1 year). o) TEM characterization of MIT@ZIF‐8 nanoparticles on day 0 and the resuspended MIT@ZIF‐8 after 1 year. p) MIT retention level in MIT@ZIF‐8 on day 0 and the resuspended MIT@ZIF‐8 after 1 year. Data for panels (d, e, h, j, l, n, and p) are mean ± standard error of the mean (s.e.m.), *n* = 3.

MIT@ZIF‐8 nanoparticles were synthesized via a one‐pot aqueous‐phase process. This method involved adding an aqueous solution of zinc nitrate into an aqueous mixture of MIT hydrochloride and 2‐MIm, followed by vigorous stirring (**Figure** **1**a; Figure S1, Supporting Information). Compared to conventional methods for synthesizing drug‐loaded ZIF‐8 nanoparticles,^[^ ^14^, ^16^ ^]^ the one‐pot aqueous‐phase method is more compatible with biomedical applications by eliminating the need for organic solvents and additional phase‐transfer processes.^[^ ^17^ ^]^ The prepared MIT@ZIF‐8 nanoparticles displayed a uniform size distribution and a polyhedral morphology, similar to those of ZIF‐8, as observed under transmission electron microscopy (TEM; Figure 1b). Dynamic light scattering (DLS) measurements indicated an average size of 183.03 ± 0.07 nm for MIT@ZIF‐8 with a polydispersity index (PDI) of 0.20, slightly larger than that of ZIF‐8 of 156.67 ± 0.71 nm with a PDI of 0.29 (Figure 1c,d). The zeta potentials of MIT@ZIF‐8 and ZIF‐8 were determined to be 25.47 ± 0.24 mV and 22.70 ± 0.59 mV, respectively (Figure 1e).

Next, various characterization methods were employed to assess the encapsulation of MIT within ZIF‐8. The loading rate of MIT in MIT@ZIF‐8 was 4.0%, and the encapsulation rate of MIT was 5.5% (Experimental Section). X‐ray powder diffraction (XRD) measurement showed that the crystal structure of ZIF‐8 remained unchanged after the incorporation of MIT (Figure 1f). X‐ray photoelectron spectroscopy (XPS) confirmed the minimal impact of MIT on the coordination of Zn^2+^ ions and 2‐MIm, as evidenced by no apparent shift between the spectra of ZIF‐8 and MIT@ZIF‐8 (Figure S2, Supporting Information). Fourier transform infrared (FTIR) spectroscopy analysis of MIT@ZIF‐8 indicated an absorption peak corresponding to the C ═ O stretching vibration of MIT (Figure 1g). These characterization results suggest that MIT is encapsulated within the porous structure of ZIF‐8.

To investigate the pH‐dependent release profile of MIT@ZIF‐8, the nanoparticles were incubated in phosphate‐buffered saline (PBS) at various pH values. Following centrifugation to eliminate the undissolved nanoparticles, the supernatant containing free MIT released from MIT@ZIF‐8 nanoparticles was detected using high‐performance liquid chromatography (HPLC) (Figure S3, Supporting Information). As shown in Figure 1h, MIT@ZIF‐8 exhibited good stability at a physiological pH of 7.4, with only 3.77% of MIT being released over a 24‐hour period, consistent with the drug release profiles of ZIF‐8 reported in previous studies.^[^ ^18^ ^]^ In contrast, when subjected to an acidic environment at pH 5.0, over 70% of MIT in MIT@ZIF‐8 nanoparticles was liberated after 24 hours (Figure 1h).

Stability is a crucial factor that affects the clinical translation and applicability of new pharmaceutical formulations.^[^ ^19^ ^]^ The stability of MIT@ZIF‐8 nanoparticles, stored as aqueous suspension at 4 °C, was first studied (Figure 1i). Over a one‐week period, MIT@ZIF‐8 nanoparticles maintained consistent size, zeta potential, and morphology (Figure 1j,k). The retention level of MIT in the nanoparticles was higher than 90%, as determined by HPLC analysis of the supernatant (Figure 1l). To evaluate the long‐term stability, MIT@ZIF‐8 nanoparticles were vacuum‐dried into powder and stored at room temperature (Figure 1m). After one‐year storage, the resuspended MIT@ZIF‐8 exhibited a size distribution and zeta potential similar to their initial values (Figure 1n). TEM analysis confirmed that the morphology of the resuspended MIT@ZIF‐8 was consistent with the original structure (Figure 1o). Notably, the MIT retention level in the resuspended MIT@ZIF‐8 was comparable to that measured immediately after synthesis (Figure 1p). These results underscore the superior stability and clinical translation potential of MIT@ZIF‐8 nanoparticles.

### 2.2 MIT@ZIF‐8 Enhances Tumor Cytotoxic Effect and Induces Pyroptosis‐Boosted ICD

**Figure 2:**
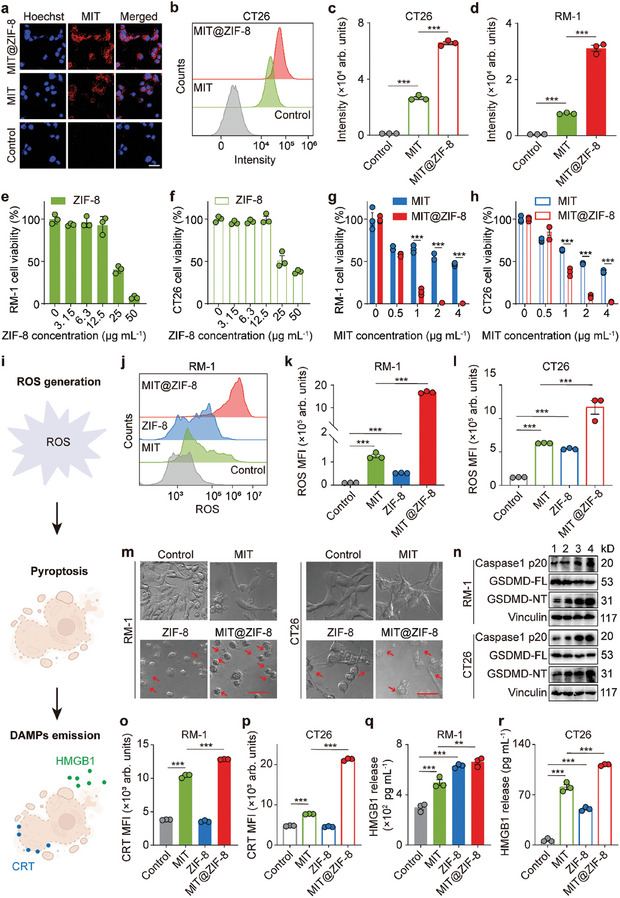
In vitro assessment of cellular drug uptake, tumor cytotoxic effect, and pyroptosis‐boosted ICD by MIT@ZIF‐8. a) CLSM images showing the intracellular distribution of MIT in CT26 cells treated with free MIT, and MIT@ZIF‐8 nanoparticles for 3 hours. Scale bar: 100 µm. b) Histogram of flow cytometry analysis of MIT uptake by CT26 cells. Quantification of MIT uptake by c) CT26 cells and d) RM‐1 cells using flow cytometry. Cell viability of e) RM‐1 and f) CT26 cells 24 hours post‐treatment with different concentrations of ZIF‐8. Cell viability of g) RM‐1 and h) CT26 cells after 24‐hour incubation with varying concentrations of MIT or MIT@ZIF‐8. i) Schematic illustration depicting ROS generation, pyroptosis induction, and enhancement of ICD by MIT@ZIF‐8. j) Histogram of flow cytometry analysis of ROS levels in RM‐1 cells. Quantification of ROS generation in k) RM‐1 cells and l) CT26 cells by flow cytometry. m) Photographs showing morphology of RM‐1 cells and CT26 cells with various treatments. Red arrows indicate cell swelling with large bubbles. Scale bar: 50 µm. n) Western blot analysis of activated caspase‐1 and GSDMD levels in RM‐1 and CT26 cells. 1: control, 2: MIT, 3: ZIF‐8, and 4: MIT@ZIF‐8. Quantification of CRT expression on the surface of o) RM‐1 cells and p) CT26 cells by flow cytometry. Quantification of HMGB1 release in q) RM‐1 cells and r) CT26 cells by ELISA. Data (c, d, e–h, k, l, and o–r) are expressed as mean ± s.e.m., *n* = 3. One‐way ANOVA (c, d, g, h, k, l, and o–r) was performed. **p < 0.01, ***p < 0.001.

To investigate the drug delivery efficiency of MIT@ZIF‐8, CT26 colon cancer cells were treated with MIT@ZIF‐8 and free MIT at the equivalent concentration, and examined using confocal laser scanning microscopy (CLSM). As shown in **Figure** **2**a, the fluorescence signals from MIT@ZIF‐8 in CT26 cells were higher than those from free MIT. Flow cytometry analysis indicated that MIT@ZIF‐8 nanoparticles increased the internalization of MIT in CT26 and RM‐1 prostate cancer cells by 2.47 folds and 3.95 folds, respectively, compared to free MIT (Figure 2b–d; Figure S4, Supporting Information). To assess the cytotoxicity of ZIF‐8 and MIT@ZIF‐8, Cell Counting Kit‐8 (CCK‐8) assay was performed to evaluate the viability of RM‐1 and CT26 cells exposed to various concentrations of ZIF‐8 and MIT@ZIF‐8. The results showed that ZIF‐8 at concentrations above 12.5 µg mL^−1^ exerted cytotoxic effects on both RM‐1 and CT26 cells (Figure 2e,f). Additionally, MIT@ZIF‐8 demonstrated an enhanced tumor inhibition effect compared to free MIT (Figure 2g,h).

Given that both ZIF‐8 and MIT can induce reactive oxygen species (ROS) production upon cellular uptake (Figure 2i),^[^ ^14^, ^20^ ^]^ we therefore evaluated the ROS‐generating ability of MIT@ZIF‐8 in RM‐1 and CT26 cells using the oxidation‐sensitive fluorescent probe 2′,7′‐ dichlorodihydrofluorescein diacetate (DCFH‐DA). As illustrated in Figure 2j–l and Figure S5 (Supporting Information), MIT@ZIF‐8 showed the highest level of ROS induction compared to ZIF‐8 and MIT, attributed to the synergistic effect of Zn^2+^ ions and MIT. Notably, RM‐1 and CT26 cells treated with MIT@ZIF‐8 exhibited the most apparent morphological changes associated with pyroptosis, such as cellular swelling and the formation of large bubbles (Figure 2m).^[^ ^21^ ^]^ Western blot analysis indicated increased levels of cleaved caspase‐1 (caspase‐1 p20) and N‐terminal fragment of cleaved GSDMD (GSDMD‐NT), as well as the cleavage of full‐length GSDMD (GSDMD‐FL) in the treated cells (Figure 2n). The binding of GSDMD‐NT with membrane phospholipids enabled pore formation on the cell membrane, facilitating the release of DAMPs to enhance ICD (Figure 2i).^[^ ^22^ ^]^ As the key indicators of ICD, the amount of CRT on the cell surface was significantly increased in MIT@ZIF‐8‐treated RM‐1 and CT26 cells (Figure 2o,p; Figure S6, Supporting Information). Additionally, the MIT@ZIF‐8 group demonstrated the highest secretion level of HMGB1 from cells (Figure 2q,r). We thus conclude that MIT@ZIF‐8 nanoparticles can effectively elicit a pyroptosis‐boosted ICD.

### 2.3 In Vivo Anti‐Tumor Effect and Immune Activation of MIT@ZIF‐8 in an Immunologically “Hot” CT26 Tumor Model

**Figure 3:**
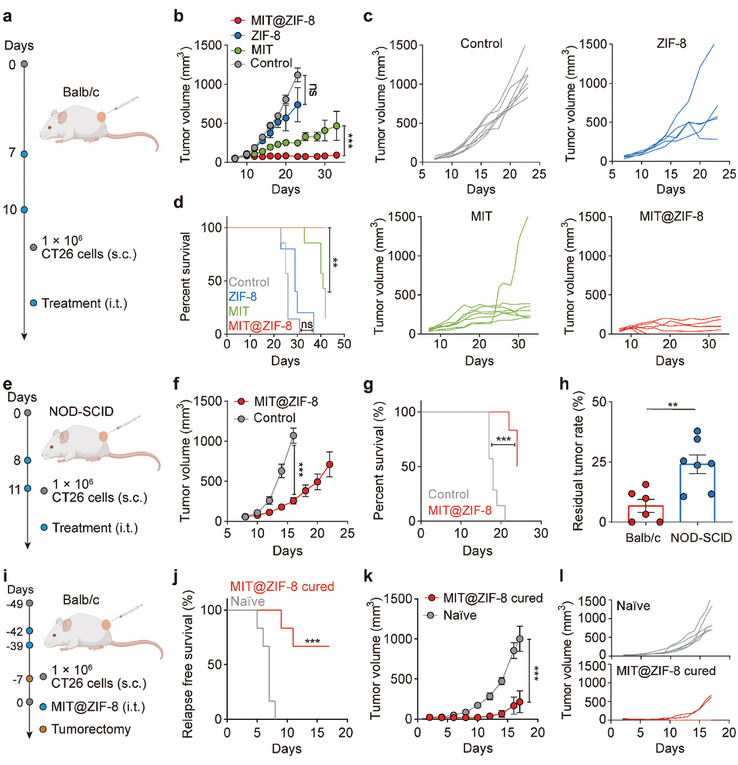
In vivo anti‐tumor efficacy and immune activation of MIT@ZIF‐8 in CT26 tumor‐bearing mice model. a) Schematic illustration for evaluating therapeutic efficacy in CT26 tumor‐bearing immunocompetent Balb/c mice. b) CT26 tumor growth curves of Balb/c mice after treatment with MIT@ZIF‐8, MIT, ZIF‐8, or 5% glucose solution (control). c) Individual tumor volume progression of Balb/c mice in MIT@ZIF‐8, MIT, ZIF‐8, and control groups, respectively. d) Survival analysis of Balb/c mice bearing CT26 tumors with different treatments. e) Schematic illustration for evaluating therapeutic efficacy in CT26 tumor‐bearing immunodeficient NOD‐SCID mice. f) CT26 tumor growth curves of NOD‐SCID mice after treatment with MIT@ZIF‐8 or 5% glucose solution (control). g) Survival analysis of NOD‐SCID mice bearing CT26 tumors with different treatments. h) Comparative residual rates of CT26 tumors in Balb/c mice and NOD‐SCID mice following MIT@ZIF‐8 treatment. i) Schematic illustration of immune activation assessment by re‐injecting CT26 tumor cells in recovered Balb/c mice upon MIT@ZIF‐8 treatment and tumorectomy. j) Kaplan‐Meier analysis depicting tumor relapse rates, k) CT26 tumor growth curves, and l) individual tumor volume progression after re‐injection of CT26 cells in recovered Balb/c mice. Data (b, f, h, and k) are presented as mean ± s.e.m. Statistical analyses include two‐way ANOVA (b, f, and k), Student's t‐test (h), log‐rank (d, g, and j). Ns: non‐significant, **p < 0.01, and ***p < 0.001.

Next, we evaluated the anti‐tumor effects of MIT@ZIF‐8 nanoparticles in an immunologically “hot” CT26 tumor model using immunocompetent, syngeneic Balb/c mice (**Figure** **3**a). To increase the drug concentration at the tumor site and reduce systemic toxicity, intratumoral injection of MIT@ZIF‐8 has been employed for in situ chemo‐immunotherapy.^[^ ^23^ ^]^ After two intratumoral administrations of therapeutic agents at a two‐day interval, MIT@ZIF‐8 (500 µg containing 20 µg MIT) and free MIT (20 µg) both led to a significant reduction in CT26 tumor growth relative to the ZIF‐8 and control groups (Figure 3b,c). Although ZIF‐8 nanoparticles can induce pyroptosis in cultured tumor cells, the administration of ZIF‐8 alone exhibited limited anti‐tumor efficacy in mice, primarily due to its insufficient induction of immune responses. Notably, MIT@ZIF‐8 showed a more pronounced inhibition of tumor growth than free MIT, with two out of six mice achieving complete remission. Additionally, MIT@ZIF‐8 treatment resulted in prolonged survival of the mice compared to those treated with free MIT (Figure 3d), suggesting that the encapsulation of MIT within ZIF‐8 improves the in vivo anti‐tumor efficacy. We should note that ZIF‐8 demonstrated some anti‐tumor efficacy; however, this effect, in terms of tumor growth inhibition and mouse survival, did not show statistical significance compared to untreated mice.

To further investigate the contribution of the immune system to the therapeutic efficacy of MIT@ZIF‐8, a CT26 tumor model in immunocompromised NOD‐SCID mice was employed (Figure 3e). Despite the immune deficiency of NOD‐SCID mice, the administration of MIT@ZIF‐8 still impeded the growth of CT26 tumor due to the intrinsic chemotherapeutic effects (Figure 3f,g). However, the tumor‐inhibitory effect of MIT@ZIF‐8 in NOD‐SCID mice was significantly compromised compared to immunocompetent Balb/c mice, as evidenced by an increase in the residual tumor rate from 6.8% in Balb/c mice to 24.0% in NOD‐SCID mice. This rate was calculated by dividing the residual tumor volume in the MIT@ZIF‐8 treatment group by the tumor volume in the control group when the average tumor volume in the control group reached 1000 mm^3^ (Figure 3h).

In a subsequent study, we examined the anti‐tumor immune activation by re‐injecting CT26 tumor cells into recovered Balb/c mice upon MIT@ZIF‐8 treatment and tumorectomy (Figure 3i). The mice that received the combined treatment showed a lower relapse rate and slower tumor growth than the naïve group (Figure 3j,k). Moreover, four out of the six cured mice did not exhibit tumor recurrence after re‐challenge with the same type of tumor cells (Figure 3l). This observation suggests the anti‐tumor memory effect induced by MIT@ZIF‐8.^[^ ^24^ ^]^

### 2.4 In Vivo Anti‐Tumor Effect and ICD Induction in an Immunologically “Cold” RM‐1 Tumor Model

**Figure 4:**
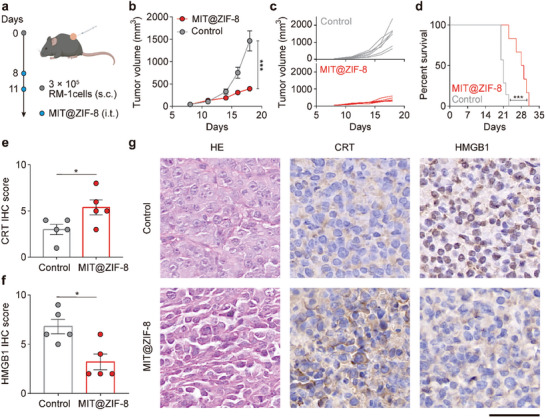
In vivo anti‐tumor activity and ICD induction of MIT@ZIF‐8 in RM‐1 tumor‐bearing mice model. a) Schematic illustration for evaluating therapeutic efficacy in RM‐1 tumor‐bearing C57BL/6J mice. b) RM‐1 tumor growth curves after treatment with MIT@ZIF‐8 or 5% glucose solution (control). c) Individual tumor volume progression in MIT@ZIF‐8 and control groups. d) Survival analysis of C57BL/6J mice bearing RM‐1 tumors with different treatments. IHC scores of e) CRT expression on the cell surface and f) HMGB1 expression in the cell nucleus of RM‐1 tumor tissues after treatment with MIT@ZIF‐8 or 5% glucose solution (control). g) Representative IHC images showing CRT and HMGB1 expression in RM‐1 tumor tissues upon different treatments. Scale bar: 50 µm. Data (b, e, and f) are mean ± s.e.m. Statistical analyses include two‐way ANOVA (b), log‐rank test (d), and Student's t‐tests (e and f). *p < 0.05, ***p < 0.001.

Having demonstrated the anti‐tumor effect of MIT@ZIF‐8 in immunologically “hot” colon cancer, we proceeded to evaluate its efficacy in immunologically “cold” prostate cancer, which often shows a poor response to immunotherapy, even in combination with chemotherapy.^[^ ^9a^ ^]^ We established an RM‐1 prostate cancer model in C57BL/6J mice via subcutaneous injection of RM‐1 cells, followed by the intratumoral administration of two doses of MIT@ZIF‐8 (500 µg containing 20 µg MIT) at a two‐day interval (**Figure** **4**a). The MIT@ZIF‐8 treatment significantly suppressed RM‐1 tumor growth (Figure 4b,c), and markedly enhanced the survival rates of C57BL/6J mice (Figure 4d). To assess the in vivo induction of ICD by MIT@ZIF‐8, RM‐1 tumor specimens were harvested eight days post treatment and analyzed by immunohistochemistry (IHC). The IHC scores indicated a substantial increase in CRT expression on the cell surface and a significant decrease in HMGB1 expression in the cell nucleus (Figure 4e–g). Given that the translocation of CRT to the cell surface and the release of HMGB1 from the nucleus are hallmarks of DAMPs, these findings suggest effective ICD induction by MIT@ZIF‐8 in immunologically “cold” prostate cancer.^[^ ^4b^ ^]^

### 2.5 Immune Activation and Reprogramming of the Tumor Microenvironment

**Figure 5:**
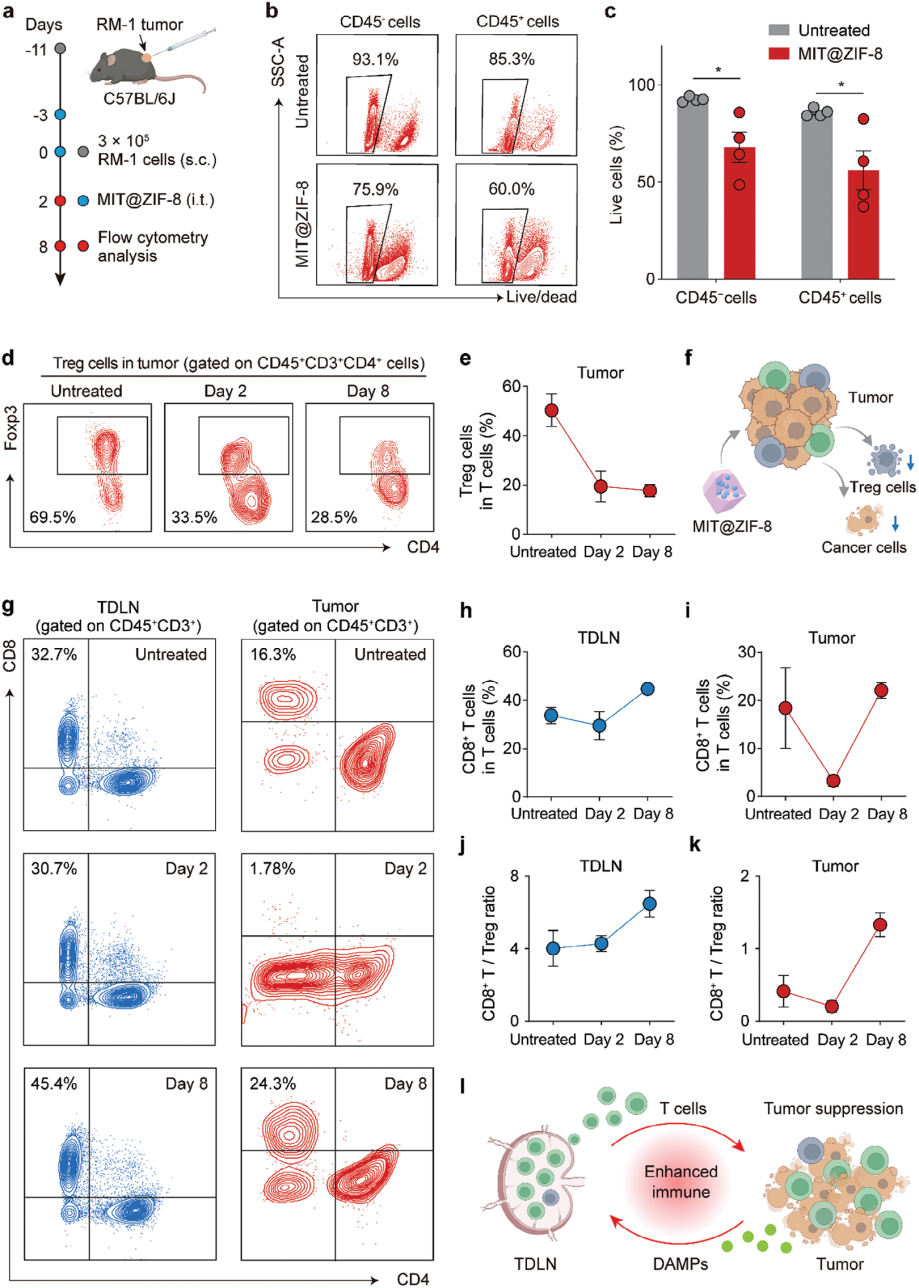
In vivo immune activation in prostate cancer by MIT@ZIF‐8 treatment. a) Schematic illustrating the experimental timeline for tumor establishment, treatment, and flow cytometry analysis. b) Flow cytometry diagrams showing viability analysis for tumor cells (CD45^−^ cells) and immune cells (CD45^+^ cells) within the tumors. c) Quantitative results of live tumor cells or live immune cells in tumors. d) Flow cytometry diagrams for Treg cells (Foxp3^+^ cells gated on CD4^+^ T cells) in tumors from untreated mice, and from mice two days and eight days after MIT@ZIF‐8 treatment. (*n* = 4 – 5). e) Quantitative results of Treg cell percentage of total T cells in tumors. f) Schematic illustrating the cancer cell killing and Treg cell depletion effects mediated by MIT@ZIF‐8. g) Flow cytometry diagrams for CD8^+^ T cells (CD8^+^CD4^−^ cells gated on CD45^+^CD3^+^ cells) in tumors from untreated mice, and from mice two days and eight days after MIT@ZIF‐8 treatment. (*n* = 4 – 5). Quantitative results of CD8^+^ T cell percentage of total T cells h) in TDLNs and i) in tumors. Ratio of CD8^+^ T cells to Treg cells j) in TDLNs, and k) in tumors from untreated mice, and from mice two days and eight days after MIT@ZIF‐8 treatment. l) Schematic illustrating enhanced anti‐tumor immunity mediated by MIT@ZIF‐8. Data (c, e, h, i, j, and k) are presented as mean ± s.e.m. Student's t‐test was conducted. *p < 0.05.

To investigate how MIT@ZIF‐8 treatment modulates the tumor microenvironment, tumor tissues and tumor‐draining lymph nodes (TDLNs) from RM‐1 tumor‐bearing C57BL/6J mice were collected at baseline (untreated) and at two days and eight days post‐treatment (**Figure** **5**a). Flow cytometry analysis indicated a significant reduction in the viability of both tumor cells (CD45^−^) and immune cells (CD45^+^) within the tumor tissues two days after MIT@ZIF‐8 treatment (Figure 5b,c, Figure S7, Supporting Information). Additionally, both the absolute abundance of immunosuppressive Treg cells and the proportion of Treg cells to total T cells in the tumor dramatically decreased two days post‐treatment, which maintained at a low level even eight days after MIT@ZIF‐8 administration (Figure 5d,e; Figure S8, Supporting Information). These results suggest that the non‐specific cytotoxicity induced by MIT@ZIF‐8 can effectively eliminate the tumor cells and pre‐existing immunosuppressive cells (Figure 5f). However, the cytotoxic effect of MIT@ZIF‐8 was confined to the tumor site, without compromising the viability of immune cells in TDLNs (Figures S9,S10, Supporting Information). The preservation of TDLN functionality, coupled with the elimination of pre‐existing dysfunctional immune cells in the tumor, facilitates the reshaping of the immune‐evasive tumor microenvironment into a more antitumorigenic state.^[^ ^25^ ^]^

Next, we assessed the MIT@ZIF‐8‐induced anti‐tumor immune activation by analyzing the amount of the cytotoxic CD8^+^ T cells and the ratio of CD8^+^ T cells to Treg cells in TDLNs and tumors (Figure 5g–k; Figures S11,S12, Supporting Information).^[^ ^26^ ^]^ As shown in Figure 5g,h, the percentage of CD8^+^ T among total T cells in TDLNs was slightly decreased two days after MIT@ZIF‐8 treatment, but then increased to 1.3 times its initial value eight days after treatment. The absolute abundance of CD8^+^ T cells in TDLNs followed a consistent trend (Figure S13, Supporting Information). Meanwhile, the percentage of Treg cells among total T cells in TDLNs was decreased by 20% at day 8 (Figure S14, Supporting Information). In comparison, the percentage of CD8^+^ T cells among total T cells in the tumors was decreased from 18.4% to 3.3% two days after treatment, attributed to the localized cytotoxic effects of MIT@ZIF‐8 (Figure 5g,i). However, a reinfiltration of CD8^+^ T cells into the tumor was observed eight days post‐treatment, with the ratio increasing to 22.1% (Figure 5g,i). The absolute abundance of CD8^+^ T cells in tumors also exhibited a similar trend (Figure S15, Supporting Information). Considering the crucial role of CD8^+^ T cells in anti‐tumor immunity and the involvement of Treg cells in immune evasion, a higher ratio of CD8^+^ T cells to Treg cells is associated with a favorable anti‐tumor immune environment.^[^ ^26^ ^]^ In our study, the ratio of CD8^+^ T cells to Treg cells in TDLNs was 4.0 in untreated mice, and increased 1.6‐fold eight days post‐treatment (Figure 5j). Within the tumors, the ratio of CD8^+^ T cells to Treg cells increased 3.2‐fold at day 8 (Figure 5k). These results suggest that MIT@ZIF‐8 treatment reprograms the tumor microenvironment to enhance anti‐tumor immunity (Figure 5l).

### 2.6 MIT@ZIF‐8 Sensitizes Prostate Cancer to Anti‐CTLA‐4

**Figure 6:**
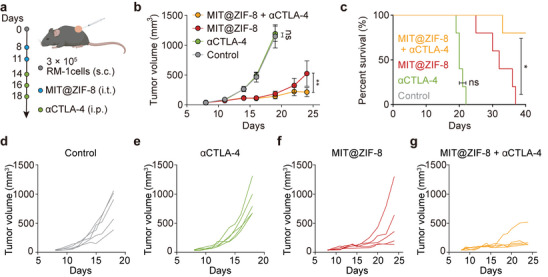
Combination of MIT@ZIF‐8 with anti‐CTLA‐4 (αCTLA‐4) for the treatment of prostate cancer. a) Schematic illustration for evaluating therapeutic efficacy of MIT@ZIF‐8 in combination with anti‐CTLA‐4. b) RM‐1 tumor growth curves after treatment with MIT@ZIF‐8 and anti‐CTLA‐4, MIT@ZIF‐8, anti‐CTLA‐4, or 5% glucose solution (control). c) Survival analysis of C57BL/6J mice bearing RM‐1 tumors with different treatments. Individual tumor volume progression in d) control group, e) anti‐CTLA‐4 group, f) MIT@ZIF‐8 group, and g) MIT@ZIF‐8 and anti‐CTLA‐4 group. Data (b) are expressed as mean ± s.e.m. Two‐way ANOVA (b) and log‐rank test (c) were conducted, Ns, non‐significant, *p < 0.05, **p < 0.01.

Anti‐CTLA‐4 immunotherapy, the first FDA‐approved ICB, has achieved sustained tumor suppression in several cancers including melanoma.^[^ ^27^ ^]^ However, its efficacy is suboptimal in tumors with low immunogenicity and minimal anti‐tumor T cell activation, such as prostate cancer.^[^ ^28^ ^]^ Given that MIT@ZIF‐8 has increased immunogenicity and induced an immune response in prostate cancer, we investigated whether MIT@ZIF‐8 could enhance the efficacy of anti‐CTLA‐4 therapy. Using RM‐1 tumor‐bearing C57BL/6J mice as a model (**Figure** **6**a), we found that standalone anti‐CTLA‐4 treatment did not inhibit RM‐1 tumor progression. In contrast, the combination of MIT@ZIF‐8 with anti‐CTLA‐4 significantly suppressed the tumor growth and prolonged mice survival (Figure 6b–g). We consider that the immunogenicity elicited by MIT@ZIF‐8, in conjunction with an increased ratio of CD8^+^ T cells, may enhance the responsiveness of prostate cancer to anti‐CTLA‐4 therapy. For prostate cancer, once resistance develops to conventional therapies like chemotherapy and hormone therapy, the available options become severely limited.^[^ ^29^ ^]^ The combination of MIT@ZIF‐8 with anti‐CTLA‐4 therapy presents a compelling alternative for the treatment of prostate cancer.

### 2.7 In Vivo Biosafety Assessment of MIT@ZIF‐8 Nanoparticles

**Figure 7:**
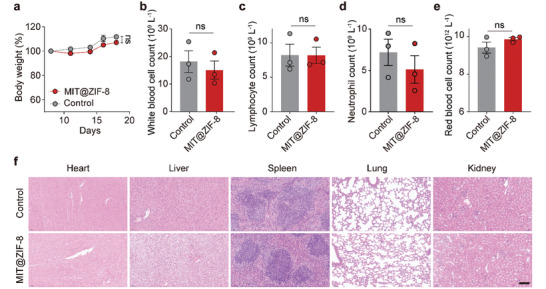
Systemic biosafety assessment of MIT@ZIF‐8 nanoparticles. a) Body weight trajectory of C57BL/6J mice during MIT@ZIF‐8 treatment. b) WBC count, c) lymphocyte count, d) neutrophil count, and e) RBC count in mice two days post‐treatment with 5% glucose solution (control) or MIT@ZIF‐8. f) Representative hematoxylin and eosin staining images of heart, liver, spleen, lung, and kidney from mice eight days post‐treatment with 5% glucose solution (control) or MIT@ZIF‐8. Scale bar: 100 µm. Data a–e) are expressed as mean ± s.e.m., *n* = 3. Student's t test (b‐e) was conducted. Ns, non‐significant.

The biosafety of MIT@ZIF‐8 nanoparticles was thoroughly investigated in a C57BL/6J murine model. Throughout the treatment period with MIT@ZIF‐8, no significant loss in body weight of the mice was observed (**Figure** **7**a). Two days after MIT@ZIF‐8 administration, a comprehensive blood analysis was conducted to evaluate the myelosuppression effect, a common side effect of MIT chemotherapy.^[^ ^30^ ^]^ As shown in Figure 7b–e, MIT@ZIF‐8 did not induce substantial changes in the levels of key blood components, including white blood cells (WBC) (Figure 7b), lymphocytes (Figure 7c), neutrophils (Figure 7d), and red blood cells (RBC) (Figure 7e), suggesting a minimal risk of hematological toxicity associated with MIT@ZIF‐8. Furthermore, histopathological examination via hematoxylin and eosin staining of major organs, including the heart, liver, spleen, lung, and kidney, demonstrated no significant morphological aberrations or tissue damage post MIT@ZIF‐8 treatment (Figure 7f). These results strongly support the good systemic safety of MIT@ZIF‐8 nanoparticles.

## 3 Conclusion

In summary, MIT@ZIF‐8 nanoparticles have been reported to enhance chemo‐immunotherapy in both immunologically “hot” colon cancer and “cold” prostate cancer, and to sensitize ICB‐unresponsive prostate cancer to anti‐CTLA‐4 immunotherapy. Distinguished from the prevalent research that employs ZIF‐8 as a drug delivery carrier, our study presents a new perspective: ZIF‐8 not only acts as a carrier to enhance tumor uptake of MIT for improved cytotoxic effect, but also triggers pyroptosis to amplify MIT‐induced ICD. The encapsulation of MIT into ZIF‐8 also confines the MIT cytotoxicity within the tumor site, preserving the functionality of TDLNs to reconfigure an anti‐tumor microenvironment. The combined effects of increased cytotoxicity, enhanced ICD, and reduced immunotoxicity significantly augment the effectiveness of chemo‐immunotherapy.

Compared to chemo‐immunotherapy regimens based on conventional systemic small molecule drugs, MIT@ZIF‐8 has shown enhanced anti‐tumor efficacy and favorable systemic safety. Although many nanomedicines can induce strong ICD after intravenous injection, these nanoparticles often require complicated synthesis procedures to incorporate multiple immunotherapeutic agents. In contrast, our MIT@ZIF‐8 consists solely of the clinically approved chemotherapeutic agent MIT and biocompatible ZIF‐8, which can be fabricated through a one‐pot aqueous‐phase process. We anticipate that MIT@ZIF‐8, a nanomedicine with highly translational potential, may advance the chemo‐immunotherapy across various cancers, including those considered as immunologically “cold”.

## 4 Experimental Section

### Materials

Zinc nitrate (Cat# 228 737) was procured from Sigma‐Aldrich (USA). 2‐methylimidazole (2‐MIm, Cat# M104839) was obtained from Aladdin Bio‐Chem Technology Co., Ltd (China). Mitoxantrone hydrochloride (MIT, Cat# M9120) was purchased from Solarbio Life Sciences (China). Cell Counting Kit (CCK)‐8 (Cat# SB‐CCK8) was acquired from Share‐Bio (China). DCFH‐DA ROS Assay Kit (Cat# S0033S) was sourced from Beyotime Biotechnology (China). Hoechst 33342 (Cat# H3570), Fixable Viability Dye eFluor™ 520 (Cat# 65‐0867‐14), and Fixable Viability Dye eFluor™ 506 (Cat# 65‐0866‐14) were obtained from Thermo Fisher Scientific Inc (USA). Anti‐calreticulin (Cat# ab92516), anti‐calreticulin‐AF488 (Cat# ab196158), and anti‐GSDMD (Cat# ab219800) were purchased from Abcam (UK). Anti‐cleaved‐caspase 1 (Cat# AF4005) was purchased from Affinity (USA). Anti‐HMGB1 (Cat# R380710) was purchased from ZEN‐Bioscience (China). Mouse HMGB1 ELISA kit (Cat# E‐EL‐M0676c) was purchased from Elabscience Bionovation Inc (China). Anti‐vinculin (Cat# 66305‐1‐Ig) was purchased from Proteintech (China). Cell Staining Buffer (Cat# 420 201), anti‐mouse CD16/32 (Cat# 156 603), anti‐CD45‐PerCP (Cat# 103 129), anti‐CD45‐PE/Cy7 (Cat# 103 113) anti‐CD3‐PE/Cy7 (Cat# 100 219), anti‐CD4‐BV650 (Cat# 100 469), anti‐CD8‐Pacific Blue (Cat# 100 728), and anti‐Foxp3‐AF488 (Cat# 126 405) were purchased from BioLegend (USA). Transcription Factor Buffer Set (Cat# 562 574) was purchased from BD Biosciences (USA). Anti‐mouse CTLA‐4 (Cat# BP0164) was purchased from BioXcell (USA).

### Preparations of ZIF‐8 and MIT@ZIF‐8

2‐MIm was dissolved in deionized water using ultrasonic agitation to a concentration of 290 mg mL^−1^. Subsequently, 4 mL of this 2‐MIm solution was diluted with an additional 4 mL of deionized water and stirred for 1 minute. Then, zinc nitrate hexahydrate was dissolved in deionized water to a concentration of 45 mg mL^−1^, and 0.5 mL of this solution was added dropwise to the 8 mL of 2‐MIm solution (145 mg mL^−1^), which was stirred vigorously at 1500 rpm for 30 minutes. For the synthesis of MIT@ZIF‐8, MIT was prepared in deionized water to a concentration of 10 mg mL^−1^ with ultrasonic assistance. Then, 0.8 mL of the MIT solution and 3.2 mL of deionized water was added to 4 mL of the undiluted 2‐MIm solution (290 mg mL^−1^) and stirred for 1 minute. 0.5 mL of zinc nitrate hexahydrate solution (45 mg mL^−1^) was added dropwise to the 8 mL of 2‐MIm (145 mg mL^−1^) and MIT (1 mg mL^−1^) mixture solution, which was stirred vigorously at 1500 rpm for 30 minutes. The resulting product was collected by centrifugation at 20 000 g for 10 minutes and washed twice with deionized water. The final product was either resuspended in aqueous solution, or subjected to vacuum drying for further characterization or experimentation.

### General Physicochemical Characterization

The morphology of the nanoparticles was characterized using a Tecnai G2 20 S‐TWIN transmission electron microscope (TEM) (FEI, USA) operating at 200 kV. Size distribution and zeta potential were measured with a dynamic light scattering (DLS) analyzer, specifically the Malvern 3000HS Zetasizer (Malvern Instruments Ltd, UK). X‐ray diffraction (XRD) patterns were obtained using a powder X‐ray diffraction instrument (Rigaku Smartlab, Japan), with measurements conducted using Cu Kα radiation over a 2θ range of 5–50° and an X‐ray power of 40 kV/200 mA. Fourier‐transform infrared (FTIR) spectroscopy of the nanoparticles was performed using a PerkinElmer Spotlight 200i instrument (PerkinElmer, USA).

### Mitoxantrone Quantification

The concentration of MIT was determined using high‐performance liquid chromatography (HPLC) with an LC‐20AT system (Shimadzu, Japan). The analysis was performed using a Wondasil C18 Wr column (Shimadzu, Japan) with mobile phases consisting of water (0.1% trifluoroacetic acid, TFA) and acetonitrile (0.1% TFA). After lysing the MIT@ZIF‐8 with hydrochloric acid, the supernatant was collected for quantification of the MIT content in MIT@ZIF‐8 using HPLC. The MIT encapsulation rate was calculated as the ratio of the weight of MIT loaded into ZIF‐8 to the total weight of MIT fed. The MIT loading rate was determined as the ratio of the mass of MIT to the mass of MIT@ZIF‐8.

### pH‐Dependent Drug Release

MIT‐loaded ZIF‐8 nanoparticles were dispersed in PBS at pH values of 5.0 and 7.4 and incubated at room temperature for 0 to 24 hours. After centrifugation (20000 g for 10 minutes), the supernatant was collected and adjusted to pH 7.4. The released MIT was quantified using HPLC. The percentage of released MIT relative to the total amount of MIT initially loaded into MIT@ZIF‐8 was calculated as the MIT release percentage.

### Cell Culture

The murine‐derived prostate cancer cell line RM‐1 and murine‐derived colon cancer cell line CT26 were procured from the Chinese Academy of Science Cell Bank (Shanghai, China). RM‐1 and CT26 cells were cultured in RPMI‐1640 medium (Gibco, C11875500BT) supplemented with 10% fetal bovine serum (FBS, Gibco, 10099141C) and 1% penicillin/streptomycin (Gibco, 15 140 122). All cell lines were maintained in a humidified incubator with 5% CO_2_ at 37 °C.

### Cellular Uptake

For confocal microscopy analysis, 2 × 10^5^ cells were seeded in confocal petri dishes and allowed to adhere. Following adherence, cells were treated with MIT and MIT@ZIF‐8 (0.2 µg mL^−1^ MIT‐equivalent) for 3 hours. After treatment, cell nuclei were stained with Hoechst 33342 (10 µg mL^−1^), and the samples were analyzed using a Zeiss 710 confocal laser scanning microscope (Zeiss, Germany). For cellular uptake quantification, after 3 hours of incubation with MIT or MIT@ZIF‐8 (0.2 µg mL^−1^ MIT‐equivalent), cells were collected, washed with Cell Staining Buffer, and the fluorescence of MIT was assessed using flow cytometry (ACEA NovoCyte, Agilent, USA) with detection in the APC channel.

### Cell Viability Assay

A total of 5000 cells were seeded in a 96‐well plate. After cell adherence, different concentrations of ZIF‐8 nanoparticles, MIT, and MIT@ZIF‐8 were added and incubated for 24 hours. Following this incubation, 10 µL of CCK‐8 reagent was added to 100 µL of complete medium in each well, and the cells were incubated at 37 °C for an additional 2 hours. Cell viability was assessed by measuring the optical density at 450 nm using a microplate reader (Synergy H1, Biotek, USA).

### ICD Detection

Cells were treated with MIT (0.6 µg mL^−1^), ZIF‐8 (15 µg mL^−1^), or MIT@ZIF‐8 (15 µg mL^−1^) for 24 hours. To assess ROS levels, cells were incubated with 10 µM DCFH‐DA in RPMI‐1640 without FBS and antibiotics for 20 minutes at 37 °C. The cells were then washed with PBS and analyzed using a flow cytometer (ACEA Novo Cyte, Agilent, USA) with detection in the FITC channel. For the detection of HMGB1 release, supernatants were collected after the 24‐hour treatment and treated by centrifugation at 5000 × g to remove cellular debris. The concentration of HMGB1 in the supernatants was measured using an ELISA following the manufacturer's instructions. To assess CRT exposure on the cell surface, cells treated for 24 hours were harvested, washed with PBS, and then incubated with anti‐calreticulin‐AF488 for 30 minutes. The expression of CRT on the cell membrane was evaluated via flow cytometry.

### Western Blot

Western blot analysis was performed following established protocols as previously described.^[^ ^31^ ^]^ Briefly, cells treated under specific conditions and durations were harvested, solubilized in lysis buffer, and heated at 100 °C for 10 minutes to denature the samples. The denatured lysates were separated by sodium dodecyl sulfate–polyacrylamide gel electrophoresis (G2043, Servicebio, China), initially running at 80 V for 30 minutes, followed by 130 V for approximately 60 minutes. Subsequently, the separated proteins were eletrotransferred onto methanol‐activated polyvinylidene fluoride (PVDF) membranes at 300 mA for 90 minutes in an ice bath. After blocking with 5% bovine serum albumin dissolved in Tris‐buffered saline with 0.1% Tween 20 (TBST) at room temperature for 60 minutes, the PVDF membranes were incubated with specific primary antibodies at 4 °C overnight. Following this incubation, the membranes were washed with TBST and then incubated with horseradish peroxidase‐conjugated secondary antibodies (ab6721 or ab205719, Abcam, UK) at room temperature for 60 minutes. After another round of washing with TBST, the blots were visualized using a chemiluminescence reagent (P0018FS, Beyotime, China).

### Animal Experiments

Six‐week‐old female Balb/c mice, 6‐week‐old female NOD‐SCID mice, and 6‐week‐old male C57BL/J mice were procured from SPF Biotechnology Co., Ltd. (China). The mice were housed under controlled conditions with a temperature of 22 ± 0.5 °C, humidity maintained at 60% ± 3%, and subjected to a 12‐hour light/dark cycle. Ethical approval for this study was obtained from the ethics committee of the National Center for Nanoscience and Technology, China.

To evaluate the anti‐tumor efficacy of MIT@ZIF‐8 in colon cancer, a subcutaneous CT26 tumor model was established by injecting 1 × 10^6^ CT26 cells into syngeneic Balb/c mice. When the tumor size reached 50 mm^3^ on day 7, the mice were randomly divided into four groups (5‐7 mice per group): control, MIT (20 µg per mouse), ZIF‐8 (500 µg per mouse), and MIT@ZIF‐8 (500 µg per mouse). Treatments were administered intratumorally on day 7 and day 10. Tumor volume was monitored and calculated using the formula: length × width^2^ / 2. The experiment continued until mice reached the predefined endpoint, which was either death or when the tumor volume reached 1500 mm^3^.

To evaluate the anti‐tumor efficacy of MIT@ZIF‐8 in an immunodeficient environment, 1 × 10^6^ CT26 cells were subcutaneously injected into NOD‐SCID mice. When the tumor size reached 50 mm^3^ on day 8, the mice were randomly divided into 2 groups (7 mice per group) and received either control or MIT@ZIF‐8 treatment (500 µg per mouse) intratumorally on day 8 and day 11. Tumor volumes were monitored throughout the experiment.

For evaluation of tumor relapse following MIT@ZIF‐8 treatment, tumorectomy was performed to completely remove residual tumor tissue. Subsequently, 1× 10^6^ CT26 cells were reinjected subcutaneously into mice previously cured with MIT@ZIF‐8 plus surgery or into MIT@ZIF‐8 treatment‐naïve mice. Tumor relapse and growth were monitored, with relapse defined as the tumor reaching 50 mm^3^. Tumorectomy procedures involved anesthesia with Zoletil and xylazine, followed by surgical excision of residual tumors using aseptic techniques. Mice were shaved, disinfected three times with 75% alcohol, draped with sterile covers, and incisions made with sterile ophthalmic scissors. After completing tumor removal, wounds were sutured with sterile sutures. Mice were allowed to recover from anesthesia before being returned to their housing.

To assess the anti‐tumor efficacy of MIT@ZIF‐8 in prostate cancer, 3 × 10^5^ RM‐1 cells were subcutaneously injected into C57BL/J mice to establish a subcutaneous RM‐1 tumor model. When the tumor size reached 50 mm^3^ on day 8, mice were randomly divided into 2 groups: control (*n* = 7) and MIT@ZIF‐8 (*n* = 6). MIT@ZIF‐8 treatment (500 µg per mouse) was administered intratumorally on days 8 and 11, with subsequent monitoring of tumor volume.

To evaluate the synergistic effects with anti‐CTLA‐4 immunotherapy, when the tumor volume reached 50 mm^3^ on day 8, mice were further divided into the following groups (*n* = 5 for each group): control, MIT@ZIF‐8, anti‐CTLA‐4, and MIT@ZIF‐8 + anti‐CTLA‐4. MIT@ZIF‐8 treatment (500 µg per mouse) was given intratumorally on days 8 and 11, while anti‐CTLA‐4 antibody (200 µg per mouse) was peritoneally administered on days 14, 16, and 18.

### Biosafety Assessment

During MIT@ZIF‐8 treatment, the body weight of mice in each group was monitored. Two days after treatment, peripheral venous blood was collected for complete blood count analysis. Eight days post‐treatment, the heart, liver, spleen, lung, and kidney tissues were harvested, processed, and embedded in paraffin for histological examination using hematoxylin and eosin (HE) staining.

### Immunohistochemistry (IHC)

IHC was conducted as previously described.^[^ ^29c^ ^]^ Briefly, the sections were deparaffinized with xylene and rehydrated through sequential immersion in absolute ethanol, 85% ethanol, and 75% ethanol. Antigen retrieval was performed using EDTA antigen repair buffer (pH 9.0) with microwave heating, after which slides were cooled to room temperature. Endogenous peroxidase activity was blocked with a 3% hydrogen peroxide solution, followed by 30 minutes of blocking with 3% BSA at room temperature. Primary antibody staining was conducted overnight at 4 °C, and secondary antibody incubation was performed at room temperature. Immunostaining was observed using DAB, and the nuclei were counterstained with hematoxylin. The sections were then dehydrated and mounted. Anti‐calreticulin (dilution 1:200) and anti‐HMGB1 (dilution 1:100) antibodies were employed for IHC staining. Intensity and percentage of positive cells were measured separately for each sample, with intensity scored as 0 (negative), 1 (weak), 2 (moderate), or 3 (strong) and percentage scored as 0 (0%), 1 (1%–25%), 2 (26%–50%), 3 (51%–75%), or 4 (76%–100%). The final IHC score was calculated as intensity score × percentage score.

### Flow Cytometry Analysis of Immune Microenvironment

Tumors and TDLNs were collected from untreated mice and from mice two days and eight days post‐treatment. Single‐cell suspensions were prepared by digesting scissored tumors with dissociation buffer (1 mg mL^−1^ Collagenase and 0.05 mg mL^−1^ DNase) or by directly dissociating TDLNs using a syringe plunger flange, followed by filtration through a 70 µm cell strainer. For tumor samples, 3 × 10^6^ cells were transferred into flow tubes for staining, while 2 × 10^6^ were used for lymph node samples. The samples were initially blocked with anti‐mouse CD16/32 at 4 °C for 10 minutes. Next, Fixable Viability Dye eFluor™ 506 and cell surface marker antibodies (anti‐CD45‐PerCP, anti‐CD3‐PE/Cy7, anti‐CD4‐BV650, and anti‐CD8‐Pacific Blue) were added and incubated at 4 °C in the dark for 30 minutes. After washing with staining buffer, Fix/Perm from the Transcription Factor Buffer Set was added and incubated in the dark at 4 °C for 50 minutes. The samples were then washed with Perm/Wash buffer, stained with anti‐Foxp3‐AF488 at 4 °C in the dark for 50 minutes, and finally washed with Perm/Wash before being resuspended in 350 µL of staining buffer for flow cytometry analysis using a BD LSRFortessa (BD, USA).

### Statistical Analysis

All statistical analyses were conducted using GraphPad Prism (version 9; GraphPad Software, La Jolla, CA, USA). Quantitative data from experiments with biological replicates were presented as mean ± standard error of mean (s.e.m.). Statistical methods included analysis of variance (ANOVA) and Student's t test. Survival curves were generated using Kapla–Meier survival analysis and compared using the log‐rank test. Statistical significance was defined as *p* < 0.05.

## Conflict of Interest

The authors declare no conflict of interest.

## Supporting information



Supporting Information

## Data Availability

The data that support the findings of this study are available in the supplementary material of this article.
