# Supplementary material for: Mitoxantrone‐Encapsulated ZIF‐8 Enhances Chemo‐Immunotherapy via Amplified Immunogenic Cell Death
Source: Adv Sci (Weinh). 2025 Feb 14;12(14):2501542. doi: 10.1002/advs.202501542 (PMC11984868; doi:10.1002/advs.202501542)
Supplement: Supplementary file 1 — Supporting Information [file ADVS-12-2501542-s001.docx]

Supporting Information

Mitoxantrone-Encapsulated ZIF-8 Enhances Chemo-immunotherapy via Amplified Immunogenic Cell Death

Junhong Li, Wenxing Lv, Ziwei Han, Yike Li, Jinqi Deng, Yanjuan Huang, Shuo Wan*, Jiashu Sun*, Bo Dai*

**Supplementary figures**


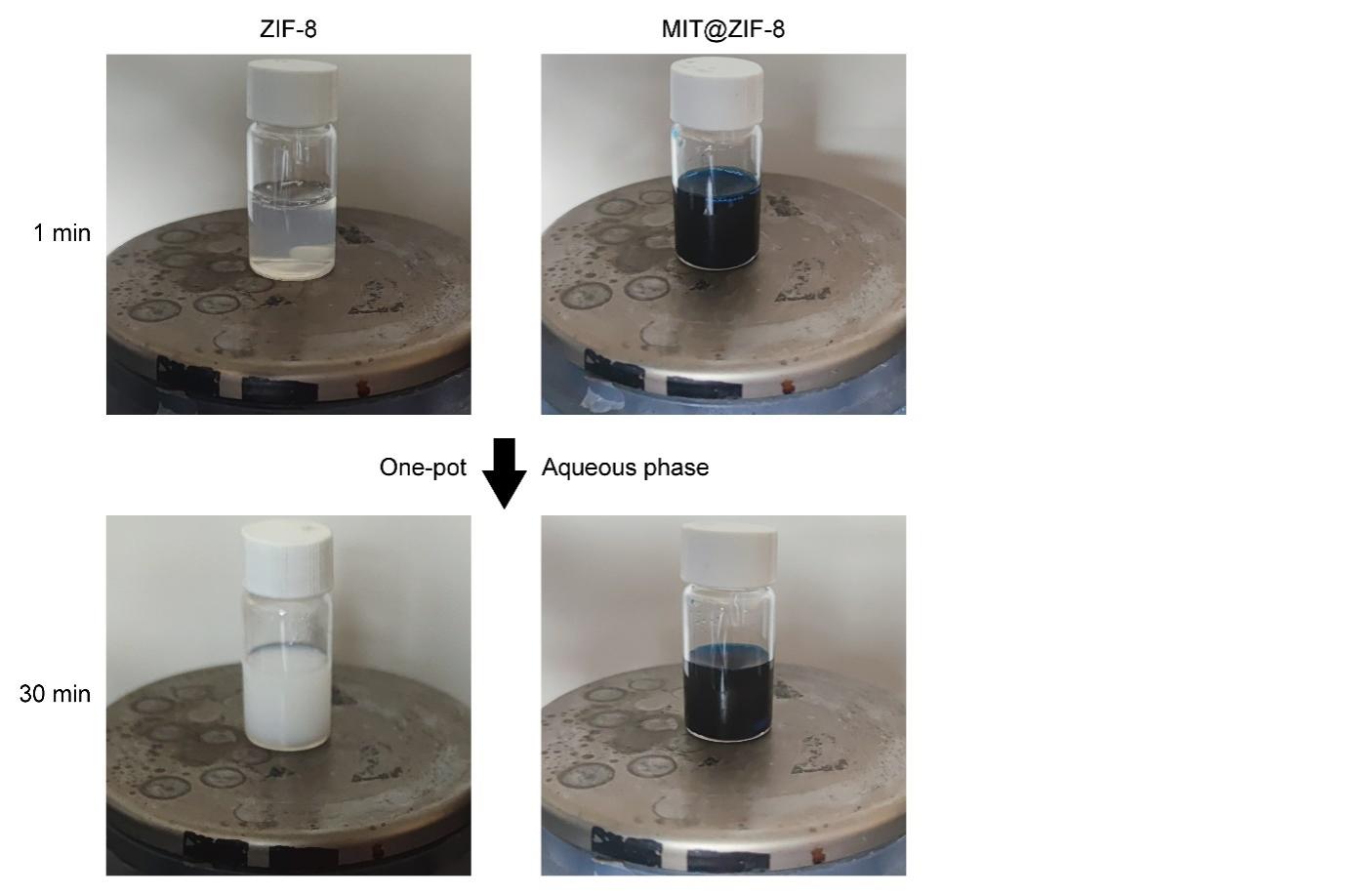


Figure S1. Photographs demonstrating the one-pot aqueous-phase synthesis of ZIF-8 and MIT@ZIF-8 nanoparticles.


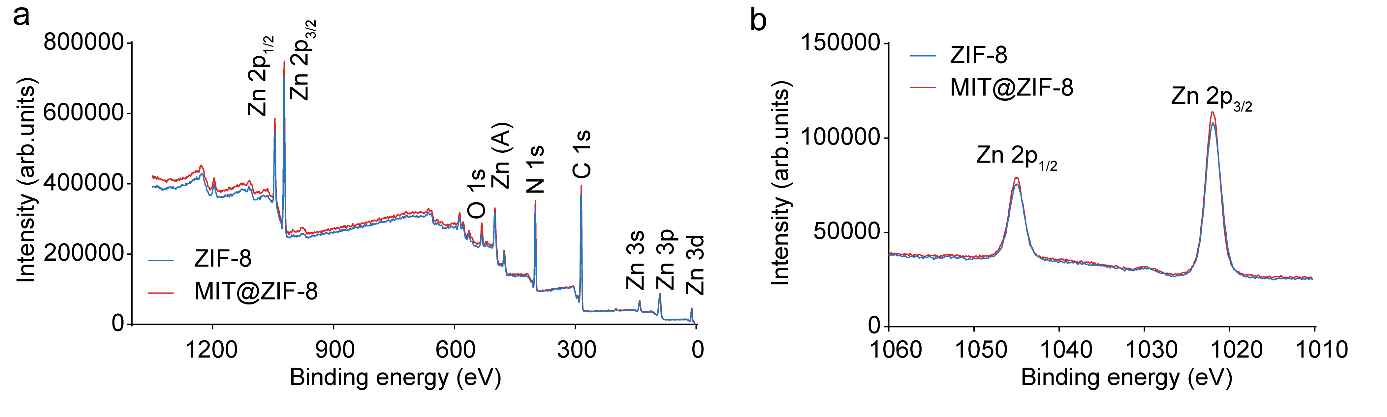


Figure S2. a) XPS spectra of ZIF-8 and MIT@ZIF-8. b) High-resolution XPS scans of Zn 2p peaks in ZIF-8 and MIT@ZIF-8.


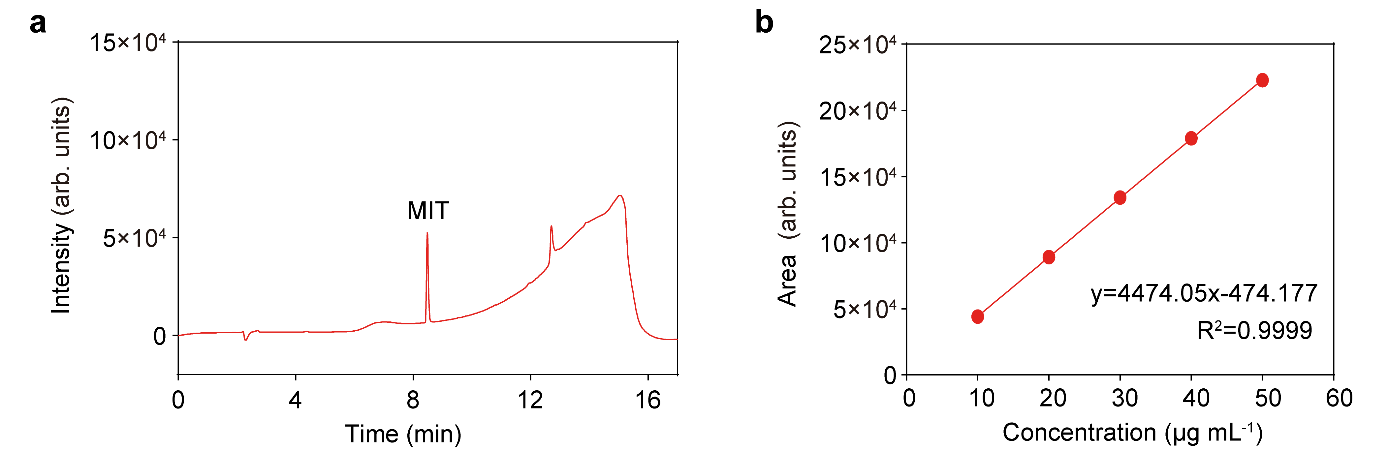


Figure S3. a) High-performance liquid chromatography (HPLC) spectrum of MIT solution, with the peak around 8.5 minutes indicating the characteristic absorption of MIT. b) A standard curve for determining MIT concentrations.


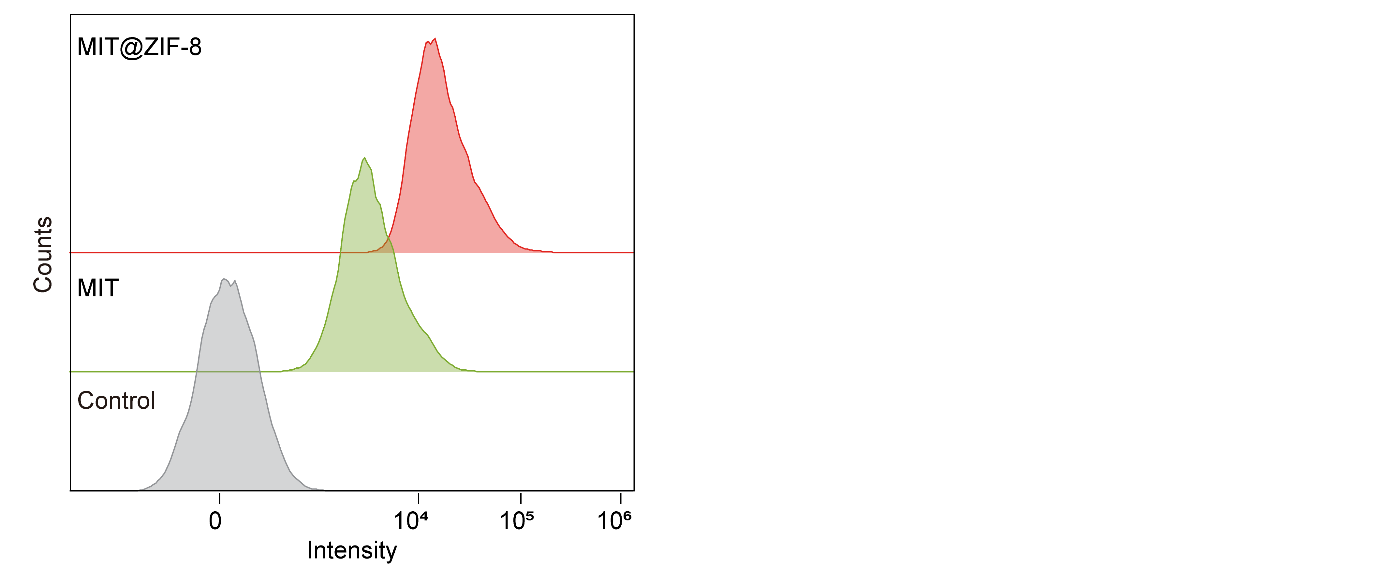


Figure S4. Histogram of flow cytometry analysis showing MIT uptake by RM-1 cells.


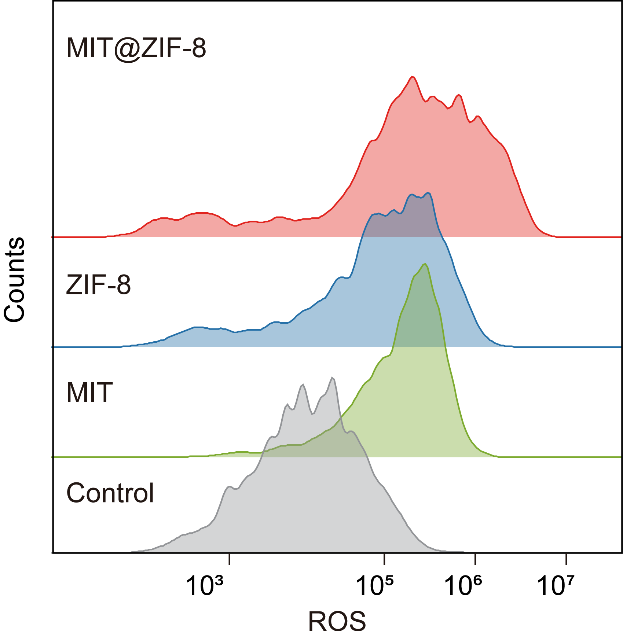


Figure S5. Histogram of flow cytometry analysis showing ROS levels in CT26 cells.


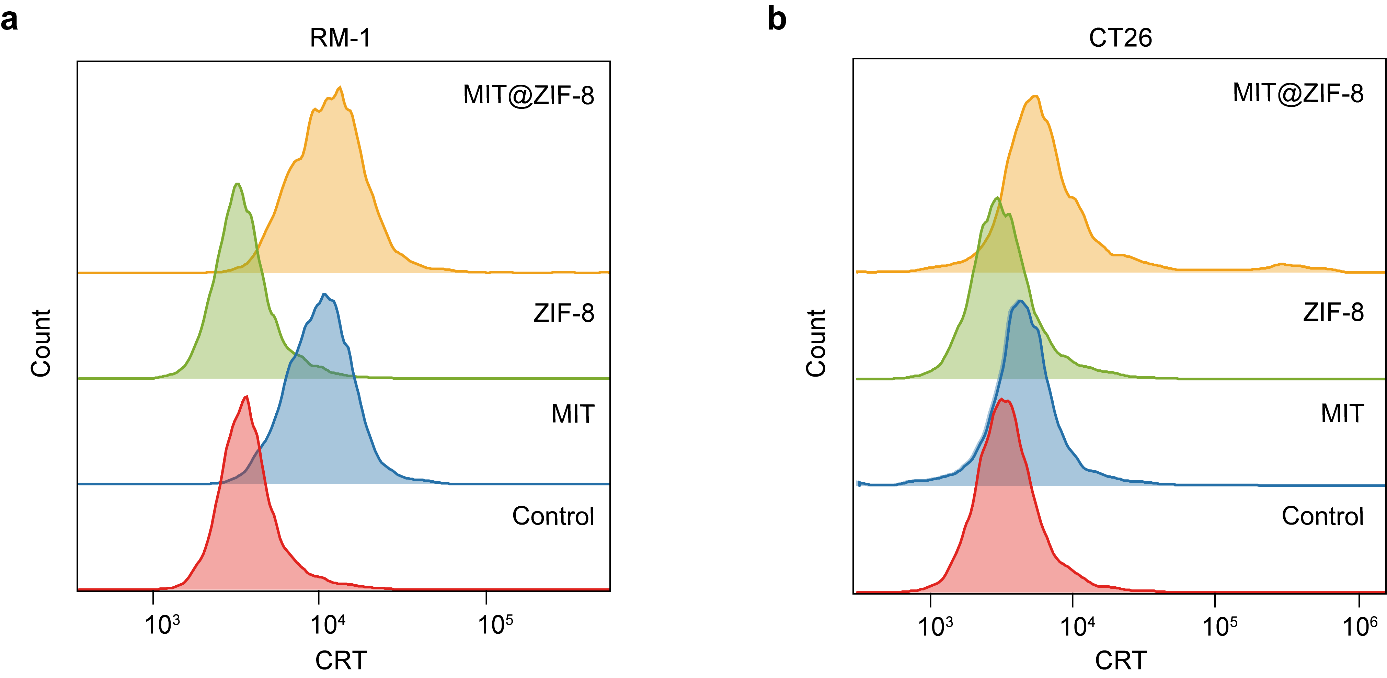


Figure S6. Histogram of flow cytometry analysis of CRT exposure on the cell surface of a) RM-1 cells and b) CT26 cells.


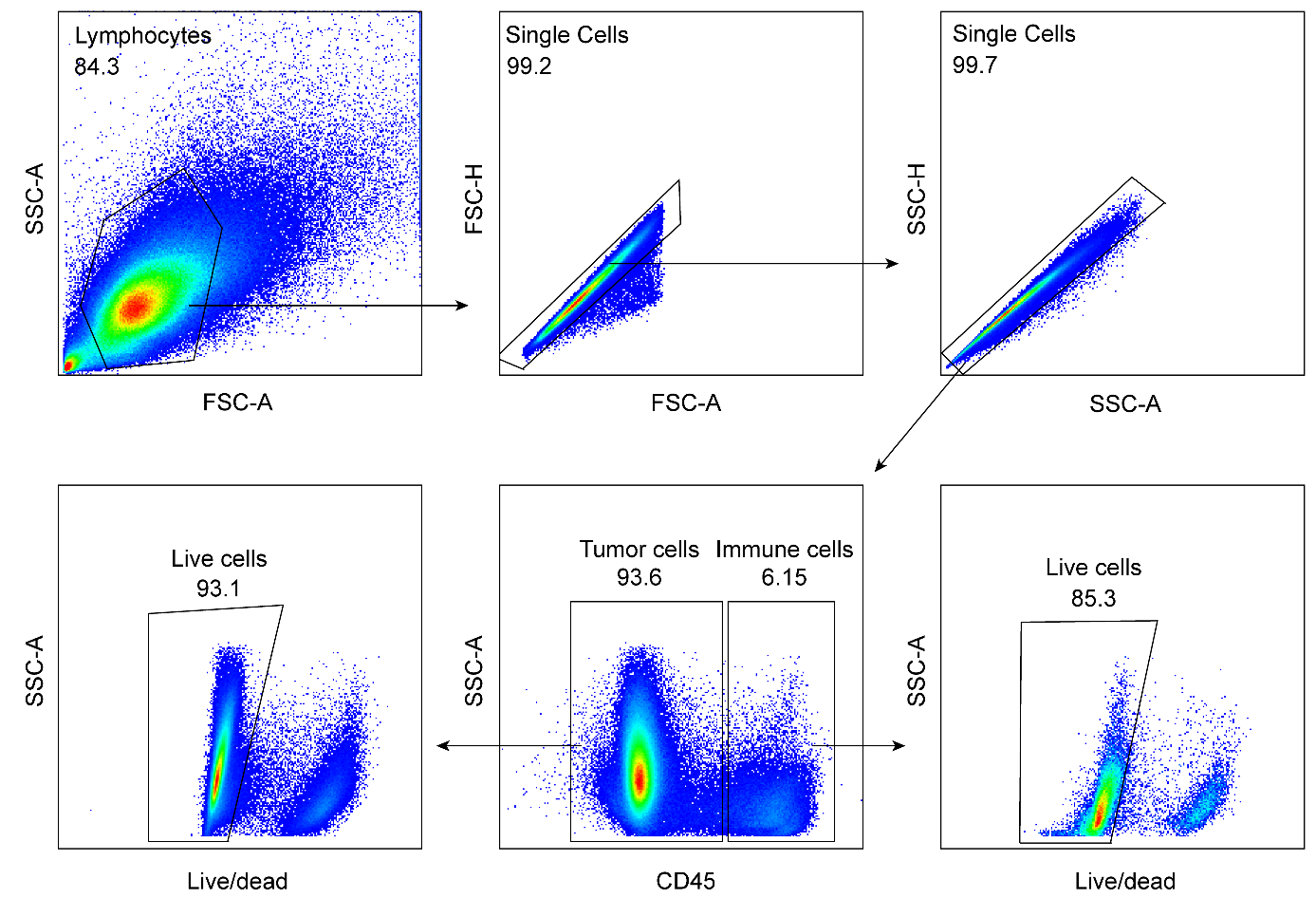


Figure S7. Flow cytometry gating strategy for analyzing cell viability of tumor cells (CD45^-^) and immune cells (CD45^+^) in RM-1 tumors using Fixable Viability Dye.


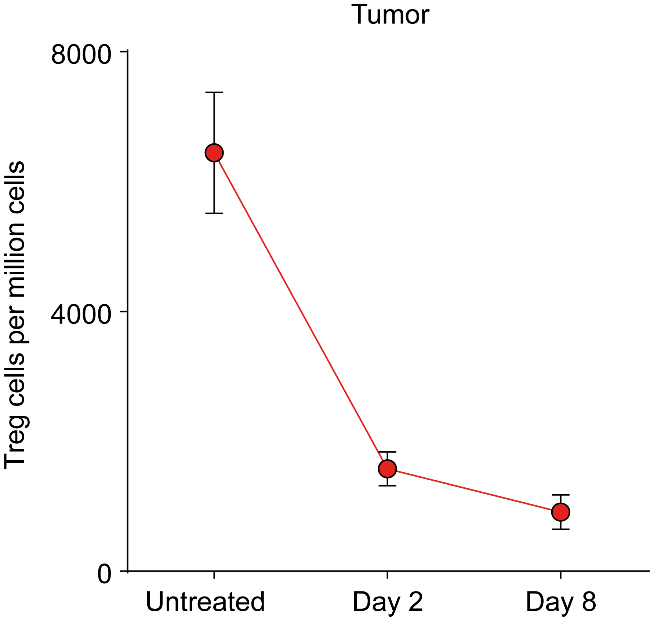


Figure S8. Quantitative analysis of the number of Treg cells per million cells in tumors (*n* = 4 - 5). Data are presented as mean ± s.e.m..


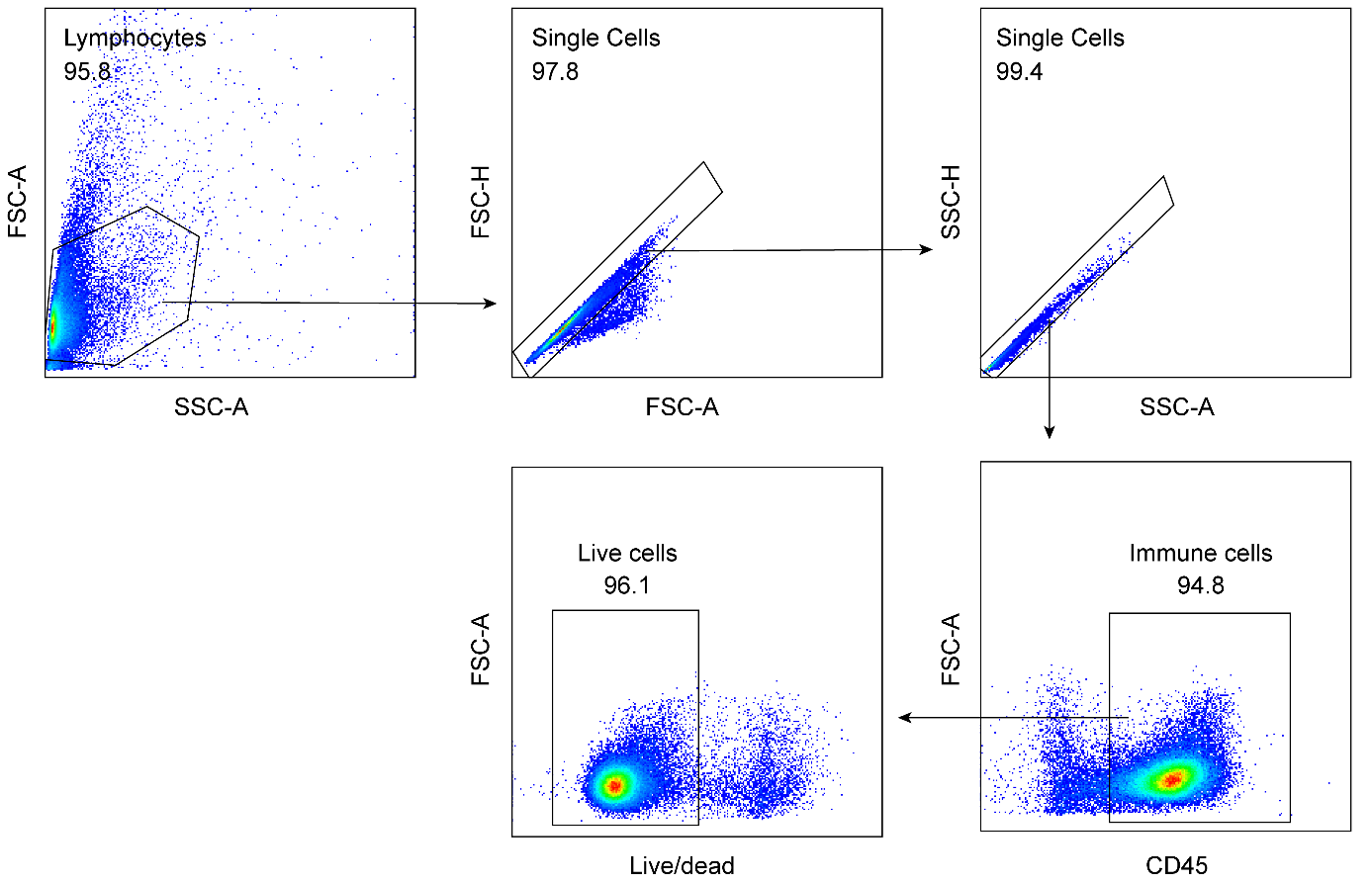


Figure S9. Flow cytometry gating strategy for analyzing cell viability of immune cells (CD45^+^) in TDLNs from RM-1 tumor-bearing mice using Fixable Viability Dye.


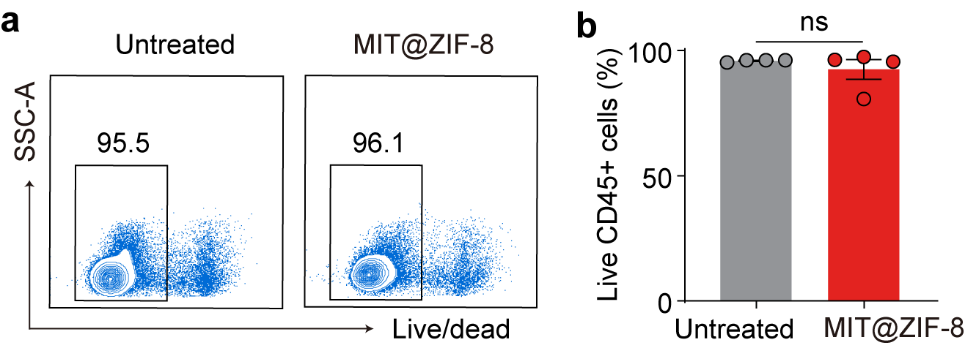


Figure S10. a) Flow cytometry diagrams showing viability analysis for immune cells in TDLNs. b) Quantitative results of live immune cells in TLDNs (*n* = 4). Data are presented as mean ± s.e.m.. Student's t-tests were conducted. Ns, non-significant.


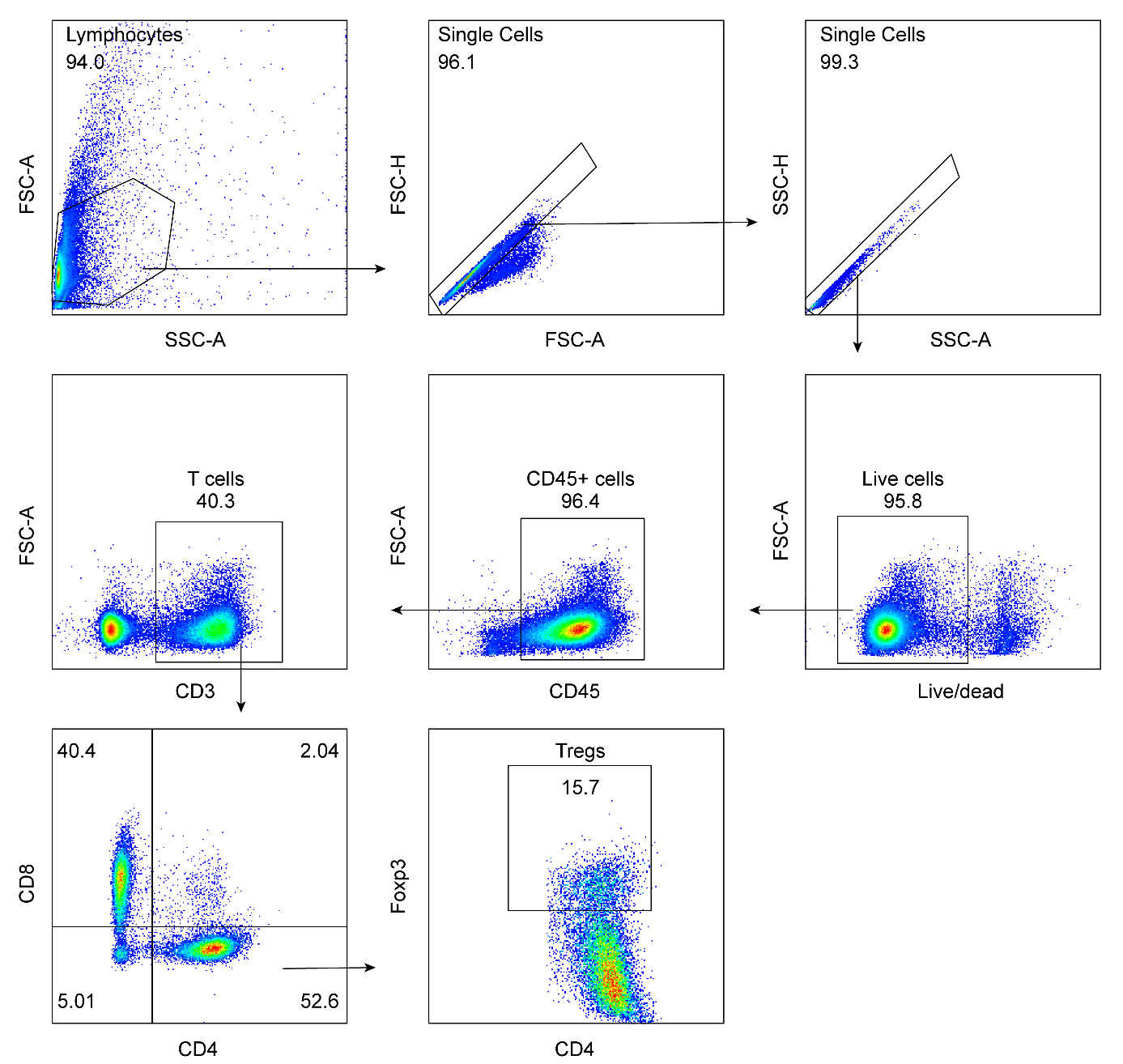


Figure S11. Flow cytometry gating strategy for defining T cells (CD45^+^ CD3^+^), CD8^+^ T cells (CD45^+^ CD3^+^ CD8^+^), and Treg cells (CD45^+^ CD3^+^ CD4^+^ Foxp3^+^) in the TDLNs. Lymphocytes were gated based on SSC-A versus FSC-A. Singlets were selected using gating on both the FSC-A versus FSC-H dot plot and the SSC-A versus SSC-H dot plot. Dead cells were excluded using Fixable Viability Dye.


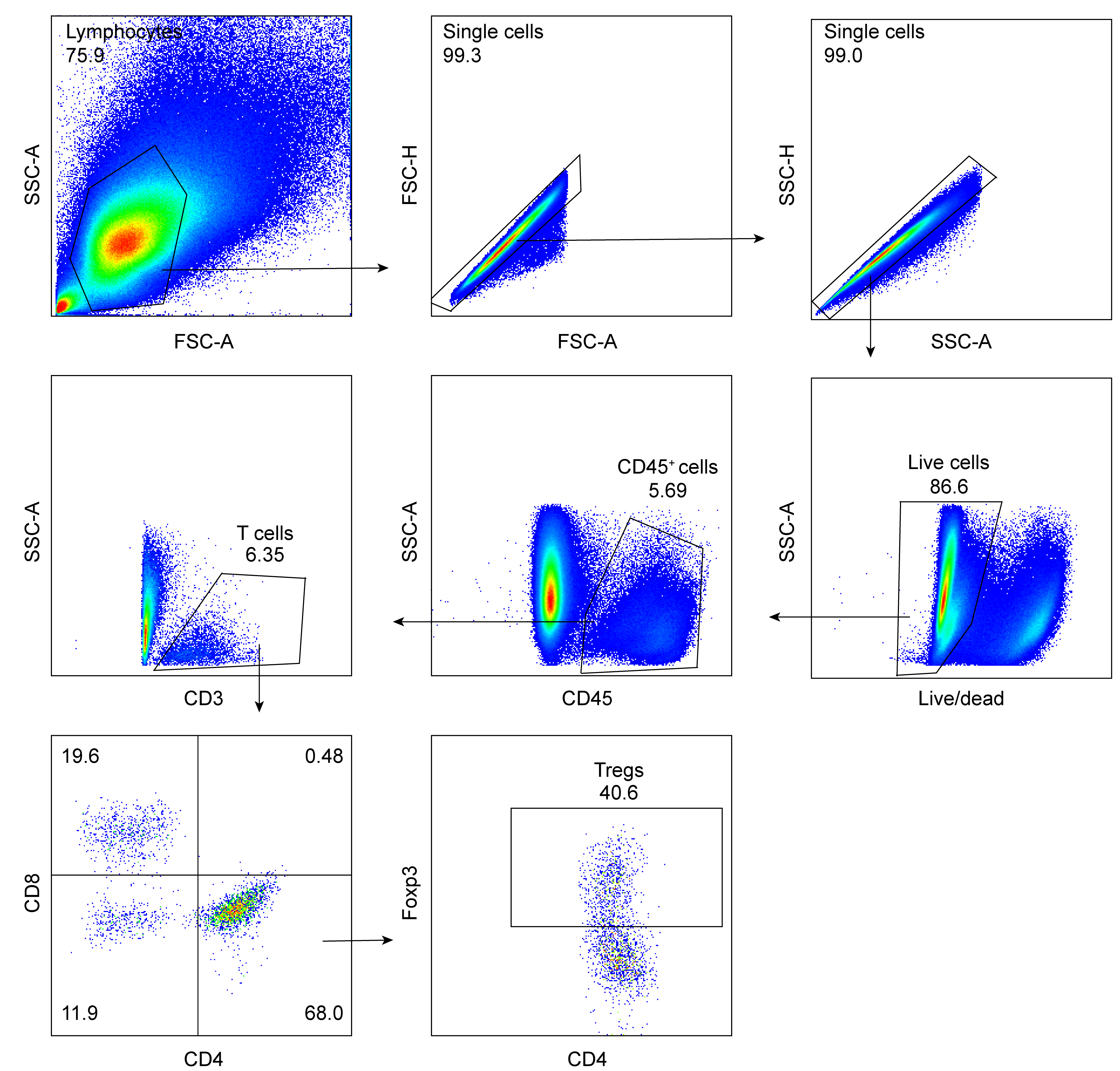


Figure S12. Flow cytometry gating strategy for defining T cells (CD45^+^ CD3^+^), CD8^+^ T cells (CD45^+^ CD3^+^ CD8^+^), and Treg cells (CD45^+^ CD3^+^ CD4^+^ Foxp3^+^) in the tumors. Lymphocytes were gated based on SSC-A versus FSC-A. Singlets were identified using gating on both the FSC-A versus FSC-H dot plot and the SSC-A versus SSC-H dot plot. Dead cells were excluded using Fixable Viability Dye.


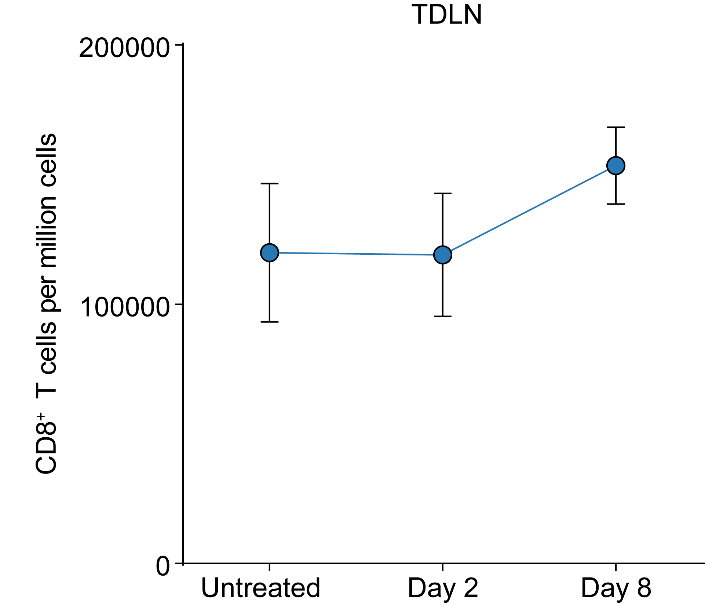


Figure S13. Quantitative analysis of the number of CD8^+^ T cells per million cells in TDLNs. (*n* = 4 - 5). Data are presented as mean ± s.e.m..


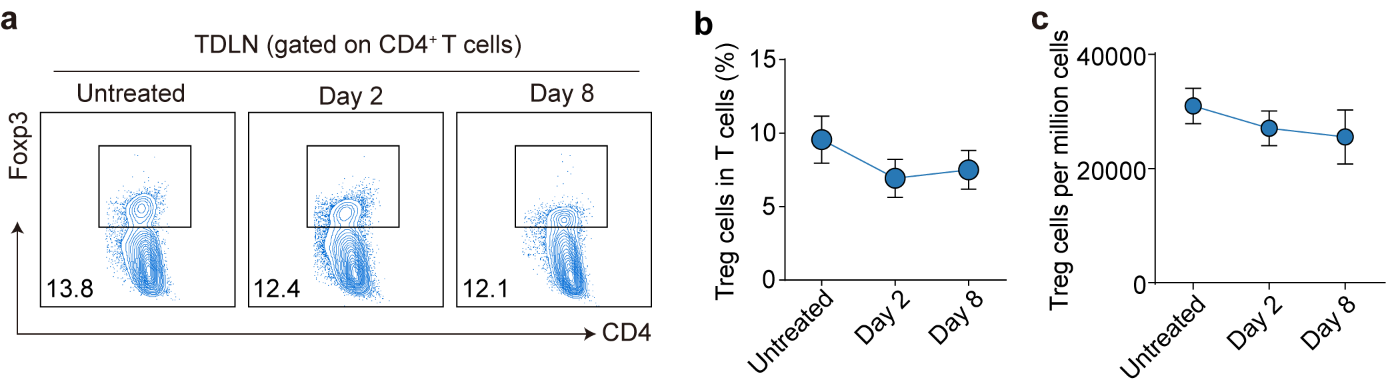


Figure S14. a) Flow cytometry diagrams for Treg cells (Foxp3^+^ cells gated on CD4^+^ T cells) in TDLNs. b) Quantitative results of Treg cell percentage of total T cells in TDLNs. c) Quantitative analysis of the number of Treg cells per million cells in TDLNs. (*n* = 4 - 5). Data are presented as mean ± s.e.m..


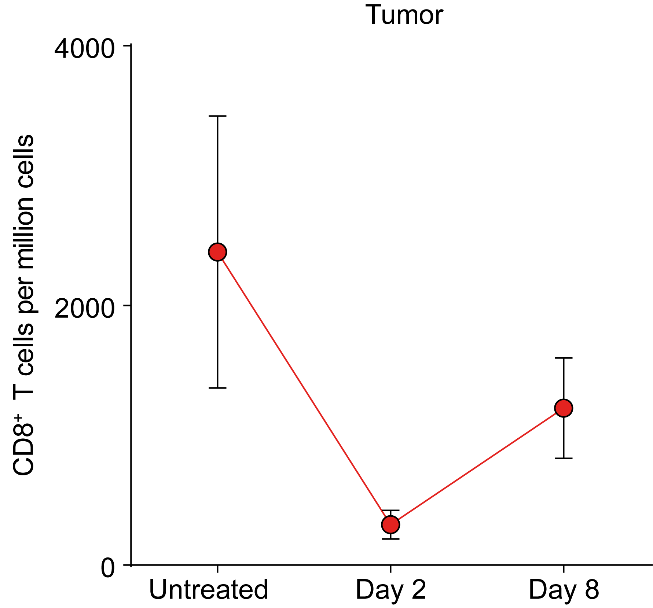


Figure S15. Quantitative analysis of the number of CD8^+^ T cells per million cells in tumors. (*n* = 4 - 5). Data are presented as mean ± s.e.m..
